# *In vivo* analysis of influenza A mRNA secondary structures identifies critical regulatory motifs

**DOI:** 10.1093/nar/gkz318

**Published:** 2019-05-04

**Authors:** Lisa Marie Simon, Edoardo Morandi, Anna Luganini, Giorgio Gribaudo, Luis Martinez-Sobrido, Douglas H Turner, Salvatore Oliviero, Danny Incarnato

**Affiliations:** 1 Dipartimento di Scienze della Vita e Biologia dei Sistemi, Università di Torino, Via Accademia Albertina 13, 10123 Torino, Italy; 2 Department of Microbiology and Immunology, University of Rochester Medical Center, 601 Elmwood Avenue, Rochester, NY 14642, USA; 3 Department of Chemistry and Center for RNA Biology, University of Rochester, Rochester, NY 14627, USA; 4 Department of Molecular Genetics, Groningen Biomolecular Sciences and Biotechnology Institute (GBB), University of Groningen, Nijenborgh 7, 9747 AG, Groningen, the Netherlands

## Abstract

The influenza A virus (IAV) is a continuous health threat to humans as well as animals due to its recurring epidemics and pandemics. The IAV genome is segmented and the eight negative-sense viral RNAs (vRNAs) are transcribed into positive sense complementary RNAs (cRNAs) and viral messenger RNAs (mRNAs) inside infected host cells. A role for the secondary structure of IAV mRNAs has been hypothesized and debated for many years, but knowledge on the structure mRNAs adopt *in vivo* is currently missing. Here we solve, for the first time, the *in vivo* secondary structure of IAV mRNAs in living infected cells. We demonstrate that, compared to the *in vitro* refolded structure, *in vivo* IAV mRNAs are less structured but exhibit specific locally stable elements. Moreover, we show that the targeted disruption of these high-confidence structured domains results in an extraordinary attenuation of IAV replicative capacity. Collectively, our data provide the first comprehensive map of the *in vivo* structural landscape of IAV mRNAs, hence providing the means for the development of new RNA-targeted antivirals.

## INTRODUCTION

Influenza A virus (IAV) represents a considerable public health threat due to its epidemic, pandemic, and zoonotic potential, causing high costs every year (1), exemplified by the tragic “Spanish” flu in 1918 which led to the death of millions of people (2). According to the World Health Organization (WHO), annual influenza epidemics cause worldwide 3–5 million cases of severe disease and up to half million deaths among high-risk groups, such as children, elderly and immunocompromised patients, with an even greater impact in developing countries. IAV belongs to the *Orthomyxoviridae* family and is a negative sense, single-stranded RNA virus. Its genome is organized into eight segments, thereby allowing for reassortment when coinfection of a host cell with different IAV subtypes occurs (3), further leading to the need for development of ever new potent antiviral agents. IAV forms three different RNA species during its life cycle in infected cells, namely the genomic minus sense viral RNAs (vRNAs), the plus sense complementary RNAs (cRNAs), serving as templates to replicate the viral genome, and the plus sense viral messenger RNAs (mRNAs) (4). The vRNAs, as well as the cRNAs, are covered with viral nucleoprotein (NP) (5,6). Within viral particles, vRNAs are associated with the viral polymerase (consisting of PA, PB1, PB2) via the panhandle structure (which poises them to be transcribed as soon as they enter the nucleus of the host cell) to form the viral ribonucleoproteins (vRNP) complexes (5). The correct packaging of all eight segments into one viral particle is believed to be conferred at least in part via structured RNA domains of vRNAs protruding from vRNPs (7–9).

RNA structure probing techniques coupled to high-throughput sequencing allow detection of structural features of RNA molecules transcriptome-wide (10). These methods provide the means to understand the previously underappreciated role RNA structure plays in the complex cellular environment. The impact that RNA structure has on regulating and fine-tuning cellular processes became particularly relevant with the extension of transcriptome-scale structure probing approaches to *in vivo* applications (11–20). Furthermore, development of mutational profiling (MaP) increased the feasibility of RNA structure probing approaches to address relevant biological questions by increasing coverage across transcripts due to read-through at probing sites, thereby omitting the need for size-selection of truncated cDNA fragments (18,21,22).

Recently, *in vivo* RNA secondary structures have further been described in the context of their specific functionalities (23–25). Zhang et al. described how mRNA structure is essential to regulate acclimation during cold shock in bacteria which is mainly regulated via Csp proteins, whose translation is in turn regulated by a structural switch in their 5′-UTRs. Beaudoin *et al.* revealed that RNA structure is dynamically regulated during development in zebrafish and showed especially 3′-UTRs to be of great importance as structures therein regulate miRNA accessibility. These studies likely mark only the beginning of an increasing research to investigate how RNA structures regulate cellular processes in greater detail. These intriguing insights further hint that RNA viruses likely regulate aspects of their replicative cycles at least in part via RNA structure.

Ever since the first genome-wide resolution of RNA structure, which modeled the *ex virio* secondary structure of the HIV-1 RNA genome (21,26) and the finding of important structural elements also in the hepatitis C virus genome (27), the interest of the scientific community in RNA structure of viruses is steadily growing. However, sparse is the knowledge about the structure that IAV mRNAs adopt and the available studies are limited to *in vitro* experiments or *in silico* predictions (28–40). Mostly, structures are described in the context of splicing of segments 7 M (M1/M2) and 8 NS (NS1/NEP). For example, Jiang *et al.* described structures at splice junctions of IAV segment 7 and 8 and showed by mutagenesis that these elements are likely functional (41). It was further shown that antisense nucleotide targeting of a structural element in segment 5 (NP) can reduce viral replication (37). However, other studies on IAV mRNAs, apart from examining structures *in vitro*, mainly remain descriptive.

Here, we present the first overview of the structure of IAV mRNAs in their native *in vivo* state during infection, as well as a thorough comparison with its structure under *in vitro* conditions. By designing mutations aimed at disrupting high-confidence structural domains, we show that the virus relies on these structural elements to promote efficient viral replication and infectivity. The work demonstrates a general pipeline for rapid investigation of *in vivo* structures for many other RNA viruses (and basically any other RNA in cells, even with low abundance). Such data has extraordinary potential to be used to design potential RNA-targeted antivirals.

## MATERIALS AND METHODS

### Infection of MDCK cells with IAV

Confluent monolayers of MDCK cells (Sigma Aldrich, #84121903-1VL) in 10 cm cell culture dishes were infected with Influenza A virus (A/Puerto Rico/8/1934(H1N1)) at a MOI of 5 in DMEM containing 0.14% BSA and 1 μg/ml TPCK-Trypsin for 6 h at 37°C. Infected cells were washed twice with PBS, and harvested by centrifugation at 524 × *g* (4°C) for 5 min. Cell pellets were snap-frozen in liquid nitrogen and kept at −80°C until use.

### *In vivo* dimethyl sulfate (DMS) probing

Cell pellets were resuspended in 1 ml of RNA probing buffer (RPB) [50 mM HEPES pH 7.9; 140 mM NaCl; 3 mM KCl] and pre-equilibrated at 37°C for 5 min. 1.76 M DMS in ethanol was added to a final concentration of 200 mM. After gentle vortexing, probing was allowed to proceed at 37°C for 2 min with moderate shaking. Reactions were quenched by addition of DTT to a final concentration of 0.7 M. Cells were collected at 2000 × *g* (4°C) for 3 min and subsequently washed once with PBS/0.7 M DTT. Cells were collected by centrifugation and total RNA extracted with TRIzol Reagent (ThermoFisher Scientific, #15596018). After phase separation, the aqueous phase was added to 1 ml of 100% ethanol and the RNA purified on RNA Clean & Concentrator-5 columns (Zymo Research, #R1016).

### DMS-MaPseq on *E. coli* rRNAs for folding parameters' calibration

For rRNA probing under *ex vivo* deproteinized conditions, a single colony of *Escherichia coli* K-12 DH10B was picked and inoculated in LB medium without antibiotics, then grown to exponential phase (OD_600_ ∼ 0.5) and 1.5 ml aliquots were collected by centrifugation at 1000 × *g* (4°C) for 5 min. Bacteria were treated identically to IAV-infected MDCK cells (see above). Deproteinized *E. coli* RNA was prepared essentially as previously described (42). Briefly, cell pellets were resuspended in 1 ml of resuspension buffer [15 mM Tris–HCl pH 8.0; 450 mM Sucrose; 8 mM EDTA], and 18 500 U of Ready-Lyse Lysozyme (Epicentre, #R1810M) were added. After incubation at 22°C for 5 min and on ice for 10 min, protoplasts were collected by centrifugation at 5000 × *g* (4°C) for 5 min. Pellets were resuspended in 120 μl Protoplast Lysis Buffer [50 mM HEPES pH 8.0; 200 mM NaCl; 5 mM MgCl_2_; 1.5% SDS]. Samples were incubated for 5 min at 22°C and for 5 min on ice, followed by addition of 30 μl SDS precipitation buffer (50 mM HEPES pH 8, 1 M KOAc, 5 mM MgCl_2_). The precipitate was removed by centrifugation at 17 000 g (4°C) for 5 min and the supernatant was subjected to gel filtration on ProbeQuant G-50 Micro Columns (GE Healthcare, #28-9034-08) pre-equilibrated three times with RNA folding buffer (RFB) [50 mM HEPES pH 8.0; 200 mM KOAc; 5 mM MgCl_2_]. The eluate was then extracted two times with phenol:chloroform:isoamyl alcohol (25:24:1, pre-equilibrated three times with RFB), and once with chloroform. 20 U of SUPERase In RNase Inhibitor (ThermoFisher Scientific, #AM2696) were added and RNA equilibrated at 37°C for 20 min prior to probing with 200 mM DMS for 2 min at 37°C with moderate shaking. The probing reactions were then quenched by DTT addition to a final concentration of 0.7 M and RNA extracted with TRIzol.

### RNA antisense purification (RAP) of IAV mRNAs

50 μg of total RNA from infected and DMS-modified cells were used for poly(A)-enrichment with Dynabeads Oligo(dT)_25_ (Thermo Fisher Scientific, #61002). The isolated poly(A) RNA was used as input for the capture of IAV mRNAs using biotinylated DNA probes (one probe for each segment with a length of 80 nt complementary to mRNA, for sequences refer to Supplementary Table S1). 100 μl of Dynabeads MyOne Streptavidin T1 beads (ThermoFisher Scientific, #65601) were washed twice with 500 μl Wash Buffer 2 [100 mM NaOH; 50 mM NaCl], twice with 500 μl Wash Buffer 3 [100 mM NaCl], and resuspended in 200 μl 2× Binding & Wash Buffer 1 [10 mM Tris–HCl pH 7.5; 2 M NaCl; 1 mM EDTA]. 5 μl of an equimolar mix of all eight probes (100 μM pool) in 200 μl water were denatured at 85°C for 3 min, then immediately placed on ice and added to the washed beads. Probes were immobilized on beads for 20 min at 37°C, with vigorous shaking. Beads were then washed twice with 500 μl 1× Binding & Wash Buffer 1 and resuspended in 100 μl RNA Binding Buffer [20 mM Tris–HCl pH 7.5; 1 M LiCl; 2 mM EDTA]. To the poly(A)-enriched RNA in 100 μl water, 100 μl RNA binding buffer were added and the sample was denatured at 70°C for 5 min and immediately placed on ice. Washed beads, supplemented with 40 U of SUPERase In RNase Inhibitor, were then added to the poly(A) RNA and binding was allowed to occur at 37°C for 2 h, with vigorous shaking. Samples were washed twice with 1 ml of Wash Buffer A (10 mM Tris–HCl pH 7.5; 150 mM LiCl; 1 mM EDTA; 0.1% SDS] and once with 1 ml Wash Buffer B [10 mM Tris–HCl pH 7.5; 150 mM LiCl; 1 mM EDTA]. Beads were resuspended in 100 μl 95% formamide and 10 mM EDTA pH 8.0, and incubated at 90°C for 10 min. Eluted IAV mRNA was separated from the beads and purified with 1 ml of TRIzol. To remove residual DNA probes, the eluate was treated with DNase I for 15 min at 37°C and further purified on RNA Clean & Concentrator-5 columns.

### Library preparation

IAV mRNA was fragmented in 64 mM Tris–HCl pH 8.3, 96 mM KCl, 3.9 mM MgCl_2_ for 8 min at 94°C and subsequently purified on RNA Clean & Concentrator-5 columns. Reverse transcription (RT) was performed using the TGIRT-III enzyme (InGex, #TGIRT50) as described previously (18). Briefly, to the 5 μl of fragmented RNA, 1 μl 10 mM dNTPs and 0.5 μl 10 μM random hexamers were added, and the mixture was heated to 70°C for 5 min and immediately placed on ice for 1 min. 2 μl of 5X RT Buffer [250 mM Tris–HCl pH 8.3; 375 mM KCl; 15 mM MgCl_2_], DTT to 5 mM, 10 U SUPERase In RNase Inhibitor, and 100 U TGIRT-III were added. Reverse transcription was then allowed to proceed at 25°C for 5 min, followed by 1.5 h at 57°C. To remove TGIRT-III from the RNA–DNA duplex, 1 μl of proteinase K was added to a final concentration of 0.1 μg/μl and the reaction incubated at 37°C for 15 min., 1 μl of a 1:2 dilution of protease inhibitor cocktail (Sigma Aldrich, #P8340) was then added to inhibit proteinase K activity. The reaction volume was then adjusted to 25 μl by adding 3 μl of 5× RT Buffer and 10 μl water. Libraries were then prepared using the TruSeq RNA Library Prep Kit v2 (Illumina, #15025063), starting from second strand synthesis.

### Denatured control samples

For denatured control samples, RNA was directly extracted from infected MDCK cell pellets with TRIzol and viral (+)RNA was purified from 75 μg total RNA using biotinylated probes as stated above, but using 150 μl Dynabeads MyOne Streptavidin T1 beads and 7.5 μl of a 100 μM equimolar probes pool. After DNase I treatment and fragmentation, samples were treated with DMS after denaturation. For that, the RNA was heated to 95°C for 1 min in a total volume of 90 μl with 55 mM HEPES pH 7.9, 4.4 mM EDTA, 55% formamide. 10 μl of 0.5 M DMS in ethanol were then added to obtain a probing concentration of 50 mM. Probing was allowed to proceed at 95°C for 1 min. Reactions were quenched by adding 200 μl RNA Binding Buffer (from RNA Clean & Concentrator-5 columns) supplemented with DTT to a final concentration of 750 mM. Samples were then purified on RNA Clean & Concentrator-5 columns. Libraries were prepared as described above (starting from RT).

### Targeted DMS-MaPseq of spliced IAV segments 7 and 8

For structure probing of spliced segments 7 and 8, infected cells were probed *in vivo* as above using DMS at a final concentration of 50 mM. 3 μg of extracted total RNA were subjected to RT as described above with slight modifications. Reactions were performed in 20 μl total reaction volume. An anchored oligo(dT) primer with overhang for subsequent PCR (Supplementary Table S2) was used at a final concentration of 50 μM. The RT reaction was allowed to proceed at 57°C for 2 h. Afterward, RNA was degraded by adding 1 μl 5 M NaOH and heating to 95°C for 3 min and subsequently purified on RNA Clean & Concentrator-5 columns. Short splice isoforms were then specifically amplified via PCR (primer sequences in Supplementary Table S2) and gel purified. The short splice isoforms were mixed equimolarly and fragmented by sonication to yield fragments in the range of 100–300 bp. Sonicated DNA was gel purified to remove residual full-length products and subjected to library preparation using NEBNext ChIP-Seq Library Prep Master Mix Set for Illumina (New England Biolabs, #E6240L) according to manufacturer's instructions, but omitting size selection.

### Targeted DMS-MaPseq of probe pairing regions

Despite DNase I treatment of captured IAV mRNA, carryover of biotinylated oligonucleotide probes occurred, resulting in lower mutation frequencies detected on probe pairing regions. To account for this, these regions were further interrogated by targeted PCR. To this end, total RNA from cells probed *in vivo* with 50 mM DMS was used. RT was performed as described above, using a 50 μM primer mix containing a specific primer for each segment. Primers annealed directly before the respective probe pairing position and contained an overhang corresponding to the standard Illumina adapter (Supplementary Table S2). cDNA was PCR-amplified and the resulting amplicons were pooled equimolarly to 1 ng/μl. Indexed adapter sequences were further added by a second PCR enrichment step.

### Targeted DMS-MaPseq of mutated domains

To confirm structure disruption in mutant viruses with respect to the wild-type (WT) structure, we interrogated these domains by targeted DMS-MaPseq analysis in cells infected with the respective IAV mutants. MDCK cells were infected as described above at a MOI of 1. Six hours post-infection, cells were harvested and probed with DMS at a final concentration of 100 mM. RT was performed as for the targeted approaches described above, using 50 μM of a specific primer (except for segment 5 for which an oligodT primer with an overhang was used, see Supplementary Table S2). PCR was then performed on the resulting cDNA using specific primers amplifying a region of ∼250–300 bp centered on the mutated domains. PCR products were pooled equimolarly and fragmented by sonication, then libraries prepared as described above for the short splice isoforms.

### *In vitro* RNA structure probing

The T7 promoter was introduced at the 5′ end of each segment via PCR using pDZ-PB2, PB1, PA, HA, NP, NA, M, NS plasmids as a template (for primers utilized see Supplementary Table S2). Segments were then *in vitro* transcribed using T7-FlashScribe kit (CellScript, #C-ASF3507). Transcription products were verified by capillary electrophoresis on a Fragment Analyzer (Advanced Analytical). For *in vitro* refolding, segments were pooled equimolarly and 240 ng of the pool were used per replicate. RNA in 15.3 μl was heated to 95°C for 2 min and immediately placed on ice for 1 min. 9 μl of 3.3× Folding Buffer [333 mM HEPES pH 7.9; 333 mM NaCl] were added and the sample incubated at 37°C for 10 min before addition of 2.7 μl of 100 mM MgCl_2_ pre-warmed to 37°C, and additional incubation at 37°C for 20 min. For probing, 1.76 M DMS in 100% ethanol was added to a final concentration of 200 mM and probing allowed to occur at 37°C for 2 min, with moderate shaking. Reactions were quenched by addition of DTT to 0.7 M and purified on RNA Clean & Concentrator-5 columns. Samples were fragmented and libraries prepared as described above.

### DMS-MaPseq data analysis

All the relevant analysis steps, from reads alignment to data normalization and structure modeling, were performed using RNA Framework (43). Briefly, sequencing reads were clipped from adapter sequences and terminal positions with Phred qualities <20 were trimmed. Alignment was performed using the *rf-map* tool and the Bowtie v2 algorithm (44), with soft-clipping enabled (parameters: *-b2 -cp -b5 5 -mp “–very-sensitive-local”*). Before proceeding to DMS-MaPseq data analysis, sequencing of an untreated IAV sample was performed in order to annotate eventual mutations with respect to the reference strain. Positions with mutations exceeding a frequency of 50% were annotated. All the subsequent analysis steps were then performed using this updated reference. Per-base mutation count and coverage were calculated using the *rf-count* tool (parameters: *-nm -r -m -mq 0 -na -md 3 -ni*). A ratiometric score was then calculated as:

\begin{equation*}\ {r_i} = \frac{{{m_i}}}{{{C_i}}}\end{equation*}

where *r_i_, m_i_*, and *C_i_* are respectively the raw reactivity, the mutation count and the coverage at position *i*. Reactivity normalization was performed as a 2-step process. First, each sample (including the denatured control) was normalized by 90% Winsorizing, in order to smooth the contribution of both over and under-reactive residues, using the *rf-norm* tool (parameters: *-sm 4 -nm 2 -rb AC -n 200*). Although median coverage was generally >1000× for each segment, we nonetheless imposed a minimum coverage of 200× per base, in order to discard lowly covered bases at segment boundaries. Briefly, each reactivity value above the 95th percentile was set to the 95th percentile and each reactivity value below the 5th percentile was set to the 5th percentile, then the reactivity at each position of the transcript was divided by the value of the 95th percentile. Reactivity data from targeted DMS-MaPseq analysis of probe pairing regions was independently Winsorized and then used to replace original RAPiD-MaPseq data. Second, both the *in vitro* and the *in vivo* datasets were normalized to the respective denatured controls:

\begin{equation*}\ {R_i} = \ min\left( {\frac{{{S_i}}}{{{D_i}}},\ 1} \right)\end{equation*}

where *R_i_, S_i_*, and *D_i_* are respectively the normalized reactivity, the sample reactivity (either *in vivo* or *in vitro*), and the denatured control reactivity at position *i*. This normalization yielded reactivity values comprised between 0 (low single-strandedness probability) and 1 (high single-strandedness probability).

Given the high correlation of all the datasets, biological replicates were combined using the *rf-combine* utility following normalization. Combined datasets were then used for all downstream analyses.

### Structure modeling

To perform structure modeling, we first used DMS-MaPseq data from deproteinized *E. coli* rRNAs to determine the optimal *slope* and *intercept* folding parameters. Fine tuning of these parameters allows penalizing (or rewarding) the formation of certain base-pairs, proportionally to the measured structural reactivity. This process is performed by predicting the reference structures (in our case *E. coli* 16S and 23S rRNAs) with different slope/intercept pairs, until the pair that gives the highest agreement to the known reference structure has been identified, a process known as *grid search* (or *jackknifing*). Grid search was performed using the *rf-jackknife* utility from the RNA Framework package (parameters: *-rp “-nlp -md 600” -x*). Optimal slope and intercept values were determined to be respectively 2.8 and −0.4 kcal mol^−1^ for the *in vivo* dataset, and 2.6 and −1 kcal mol^−1^ for the *in vitro* dataset.

Structure modeling was performed using the *rf-fold* utility from the RNA Framework package (which implements a windowed folding approach similar to the one previously employed to infer the HIV-1 genome structure (21)), and the ViennaRNA Package 2.0 (45), using the aforementioned slope/intercept value pairs (parameters: *-g -md 600 -nlp -w -pw 800 -fw 1000 -pk -km 2 -kw 600 -ko 200 -dp -sh -sl <slope> -in <intercept>*).

#### Prediction of pseudoknotted base-pairs

During the first stage, each viral segment was folded in 600 nt sliding windows, slid in 100 nt increments. DMS-MaPseq data was incorporated into the prediction, using the previously determined slope/intercept pairs. Three additional folds were computed at the 5′ and 3′ ends of each segment to increase sampling of terminal regions. Pseudoknots were required to occur in >50% of the windows containing the involved base-pairs, and to have an average DMS reactivity < 0.7 in order to be retained.

#### Partition function modeling

Partition function folding was calculated for each viral segment in 800 nt sliding windows, slid in 200 nt increments. The maximum base-pairing distance was set to 500 nt. DMS-MaPseq data was incorporated into the prediction, using the previously determined slope/intercept pairs. Three additional folds were computed at the 5′ and 3′ ends of each segment to increase sampling of terminal regions. 100 nt were trimmed from both ends of each window to avoid terminal biases. Base-pairs with >0.99 probability of formation were retained. During this stage, the Shannon entropy at each position of the transcript was calculated as:

\begin{equation*}\ {S_i} = \ - \mathop \sum \limits_{j\ = \ 1}^J {p_{i,j}}\ {\log_{10}}{p_{i,j}}\ \end{equation*}

where *S_i_* and *p_i,j_* are respectively the Shannon entropy at position *i* of the transcript and the probability of *i* being base-paired with *j* (with *j* ∈ *J*, where *J* is the set of all the potential pairing partners of *i*).

#### Minimum free energy modeling

Minimum free energy folding was performed for each viral segment in 1000 nt sliding windows, slid in 200 nt increments. The maximum base-pairing distance was set to 500 nt. DMS-MaPseq data was incorporated into the prediction, using the previously determined slope/intercept pairs. Base-pairs retained during the two previous stages were incorporated as hard-constraints in the prediction. Five additional folds were computed at the 5′ and 3′ ends of each segment to increase sampling of terminal regions. Base-pairs present in more than 50% of the windows in which they could have appeared were retained, yielding the final Minimum Expected Accuracy structure (MEA). Structure models were plotted using VARNA (46).

### Assessing conservation of helices

To assess helices' conservation, we obtained all the full-length sequences available in the NCBI Influenza Virus Database (https://www.ncbi.nlm.nih.gov/genomes/FLU/). This resulted in a total of: 14,276 sequences for segment 1, PB2 (5,050 from H1N1/H5N1); 14,619 sequences for segment 2, PB1 (5,088 from H1N1/H5N1); 14,617 sequences for segment 3, PA (5,172 from H1N1/H5N1); 11,507 sequences for segment 4, HA (3,798 from H1N1/H5N1); 13,253 sequences for segment 5, NP (4,630 from H1N1/H5N1); 23,773 sequences for segment 6, NA (9,243 from H1N1/H5N1); 12,514 sequences for segment 7, M (4,371 from H1N1/H5N1); and 29,272 sequences for segment 8, NS (11,834 from H1N1/H5N1). Identical sequences were collapsed. From each sequence, the untranslated regions (UTRs) and the coding sequences (CDS) were extracted and independently aligned using different approaches. UTRs were aligned using the Clustal Omega algorithm (47). CDSs were first translated, aligned using the Muscle algorithm (48) and then converted back to nucleotides. UTR/CDS alignments were then chained. For each predicted base-pair in our MEA structure, we then calculated a conservation frequency by counting the number of sequences in which the base-pair was maintained, divided by the total number of aligned sequences.

### Ranking of IAV RNA structural motifs

In order to prioritize IAV mRNA structural motifs more likely to have an impact on the fitness of IAV, we empirically devised a scoring scheme that rewarded more those motifs having: (i) high probability of formation (low Shannon entropy) both *in vivo* and *in vitro*, (ii) high conservation and (iii) long helices (larger domains). A score was calculated for each motif according to the formula:

\begin{equation*}S = {P_{in\;vivo}} \cdot {P_{in\;vitro}} \cdot C \cdot {\log_2}\left( {\frac{L}{2}} \right)\end{equation*}

where *S, P_in vivo_, P_in vitro_, C* and *L* are respectively the score, the *in vivo* and *in vitro* formation probabilities, the conservation and the number of base-pairs of the structural motif. Multi-branched loops were split into their individual helix components and evaluated separately. For each segment, the top-scoring motif was selected for further validation.

### Design of structure-disrupting mutants

To design structure-disrupting mutants that did not alter the underlying amino acid sequence, we developed a tool that iteratively replaced each codon within a given RNA structural motif with synonymous codons (avoiding rare codons). A maximum of three codons were allowed to be simultaneously mutated. If the motif was predicted to be part of a larger domain *in vitro*, mutations were also allowed to occur on ±10 nt surrounding the motif. After each mutation, the algorithm evaluated the base-pairing distance between the mutated and WT structures, as well as the probability of still observing the WT structure in the Boltzmann ensemble. The set of mutations maximizing the base-pairing distance and minimizing the probability of formation of the WT structure was chosen. This tool will be introduced in the next RNA Framework release. Positions of introduced mutations have been detailed in Supplementary Table S5.

### Recombinant IAV production

Bidirectional pDZ plasmids containing the respective mutated viral segments were produced using QuikChange XL Site-Directed Mutagenesis Kit (Agilent, #200516-5) in up to three successive rounds of mutagenesis. To this end, segments were first sub-cloned into pCR-Blunt II-TOPO (ThermoFisher Scientific, #K280002) using the *KpnI* restriction enzyme, in order to facilitate mutagenesis (pDZ plasmid is very GC-rich). After mutagenesis, segment sequences were checked by Sanger sequencing. Mutated segments were cloned back into the *KpnI*-digested pDZ plasmid and the correct orientation of the inserts was confirmed by PCR screening. Mutant IAVs were produced essentially as previously described (49), with slight modifications. Briefly, co-cultures of 5 × 10^5^ MDCK and 5 × 10^5^ HEK 293FT (ThermoFisher Scientific, #R70007) cells per well (six-well plate format) were plated the day before transfection. 1 μg of each pDZ plasmid was transfected using Lipofectamine 2000 (ThermoFisher Scientific, # 11668027). After 16–24 h, transfection medium was changed to viral infection medium (VIM) [DMEM; 1% PenStrep; 0.3% BSA; 1 μg/ml TPCK-trypsin (Sigma, #T-1426)]. 48 h after changing the medium, the tissue culture supernatants were centrifuged at 17 000 × *g* (room temperature) for 1 min and 500 μl of supernatant were used to infect 1 × 10^6^ MDCK cells (six-well plate format). Cells were then incubated for 48 or 72 h in VIM, until a cytopathic effect was observed. Viruses were then plaque purified and the viral titers were determined by plaque assay (see below).

### Plaque assay

Confluent monolayers of MDCK cells (2.4 × 10^5^ cells/well, 24-well plate format, triplicate) were seeded the day prior to infection. For each virus assayed, 10-fold serial dilutions (10^−3^ to 10^−6^) were prepared. Every dilution was assayed in triplicate. For infection, cells were washed twice with PBS and 150 μl of each viral dilution were added. Viral absorption was allowed to occur for 1 h at 37°C, vasculating the plate every 12 min. After viral absorption, cells were overlaid with 750 μl VIM with a final concentration of 0.7% Avicel RC 581 (FMC BioPolymer). Infection was allowed to proceed for 2 days at 37°C. Subsequently, cells were fixed for 1 h at room temperature by adding 500 μl of 4% paraformaldehyde. The supernatant was then removed and cells stained for 40 min with 1% crystal violet in PBS. Crystal violet was removed and cells carefully washed with water. Plaques were counted and PFU/ml were calculated.

### IAV multi-cycle growth kinetics

Virus multi-cycle growth kinetics were determined essentially as described (41). Briefly, confluent monolayers of MDCK cells (3.5 × 10^5^ cells/well, 12-well plate format, triplicate) were infected with the respective IAV (WT or mutant) at a MOI 1 × 10^−4^. Viral absorption was allowed to occur at 37°C in VIM for 1 h, vasculating every 12 min. Afterward, cells were washed once with PBS and overlaid with 800 μl VIM. The infection was allowed to proceed for 14 h, whereupon the supernatant was cleared by centrifugation at 17,000 × *g* (at room temperature) for 1 min. The supernatant was then subjected to plaque assay as described above to determine PFU/ml. *P*-values were calculated by Welch's *t*-test statistics.

### IAV *in vitro* evolution

Confluent monolayers of MDCK cells (3.5 × 10^5^ cells/well, 12-well plate format) were infected with the respective IAV (WT or mutants) at a MOI of 0.1 on day 1. Viral absorption was allowed to occur at 37°C in VIM for 1 h, vasculating every 12 min. Afterward, respective IAVs were removed and cells were overlaid with 800 μl VIM. Every 24 h 220 μl of supernatant were used to infect new confluent MDCK cells for a total of 12 passages. An aliquot of supernatant was saved at each passage and viral RNA was extracted in TRIzol. For analysis of mutations, extracted RNA from passages 0, 6 and 12 was reverse transcribed with Superscript II according to manufacturer's instructions (Invitrogen, #18064) using specific primers for the mutated domains (2 primers per segment were used to amplify (+) as well as (–) sense RNA of IAV) and domains were then specifically PCR amplified (see Supplementary Table S2 for primer sequences). PCR products were used for library preparation analogously to short splice isoforms.

## RESULTS

### RAPiD-MaPseq enables targeted *in vivo* RNA structure mapping of low-abundance IAV mRNAs

**Figure 1.**
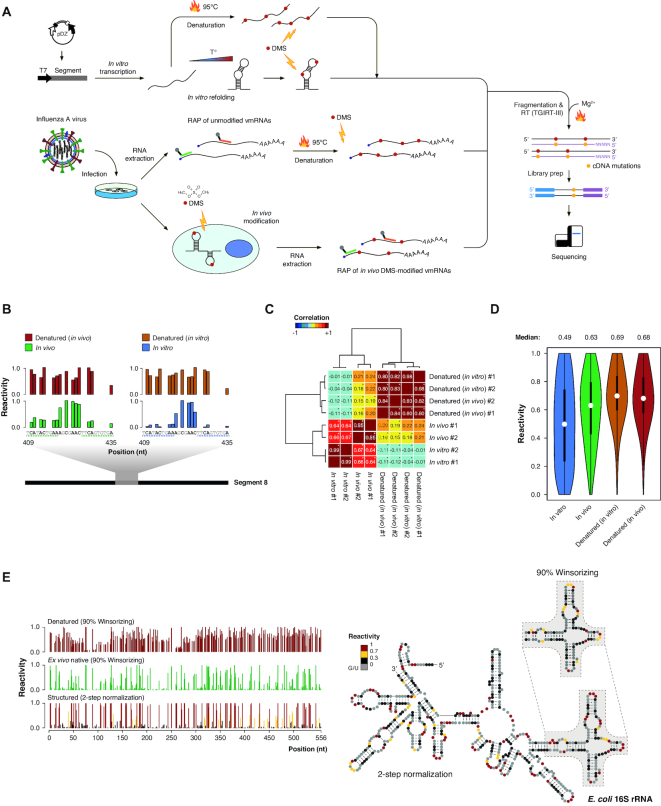
Summary of experimental conditions and data normalization. (**A**) Schematic of the experimental workflow. (**B**) Sample DMS-MaPseq reactivities from a hairpin-forming region of IAV segment 8, NS (nt 409–435). The two halves of the helix (indicated by dashed lines) have lower DMS reactivities compared to the loop region. (**C**) Heatmap of pairwise Pearson correlations for all IAV DMS-MaPseq datasets. Significant correlations (*P*-value < 0.05) are denoted by a star in the upper-right corner. (**D**) Violin plot of normalized (90% Winsorizing) DMS reactivities across IAV transcriptome. The median of each distribution is indicated. (**E**) (*left*) Snapshot of 90% Winsorizing and 2-step normalized DMS reactivities on the first 550 nt of *E. coli* 16S rRNA under both denaturing and native deproteinized *ex vivo* conditions. (*right*) 2-step normalized DMS reactivities superimposed onto the phylogenetic-derived secondary structure of the *E. coli* 16S rRNA. The inlay shows superimposition of 90% Winsorizing-normalized reactivity values of the boxed domain for comparison.

To investigate IAV mRNA structures in the context of infected cells, we here developed RNA antisense purification combined with dimethyl sulfate (DMS) mutational profiling (RAPiD-MaPseq), an approach that couples *in vivo* DMS probing with consecutive oligo-dT and biotinylated antisense oligonucleotide-mediated capture of target mRNAs, thus exploiting the high affinity and specificity of the biotin-streptavidin interaction to achieve over 13-fold enrichment of IAV mRNAs (Supplementary Figure S1A). DMS is an alkylating reagent, able to readily permeate cell membranes and modify unpaired A and C residues with fast kinetics (10). This approach provides the unique opportunity of allowing to investigate the secondary structure of low-abundance RNA species in living cells. Thus, we exploited RAPiD-MaPseq to query the structure adopted by Influenza A/Puerto Rico/8/1934(H1N1) IAV mRNAs during infection of living cells (Figure 1A). We further generated an *in vitro* dataset by *in vitro* transcription and refolding, in order to both identify thermodynamically favored RNA structural motifs and characterize IAV mRNAs structural differences between *in vivo* and *in vitro* conditions. As it has been previously shown that context-specific biases exist and that not all residues have the same propensity to react with DMS (12), a denatured control sample was also obtained by treating IAV mRNAs with DMS under denaturing conditions, hence providing a measure of the maximum possible reactivity for each residue (Figure 1B).

Two additional datasets were generated. To account for the eventual carryover of RAP capture probes, often causing lower mutation frequencies on probe pairing regions of IAV mRNAs, structural signal on these regions was further measured by targeted DMS-MaPseq (Supplementary Figure S1B). Moreover, as IAV segments 7 and 8 are known to undergo at least one alternative splicing event (resulting in two shorter splice isoforms), we specifically interrogated these shorter variants by targeted DMS-MaPseq analysis.

Successful isolation of IAV mRNAs was confirmed by the drop of sequencing coverage observed right before the poly(U) stretches used by the viral RNA-dependent RNA polymerase complex as a template for the synthesis of poly(A) tails of IAV mRNAs (Supplementary Figure S1C). Reverse transcription of the DMS-modified RNA was carried out using the TGIRT-III reverse transcriptase, which is able to read through DMS alkylated residues with high efficiency (18). Accordingly, between 86% and 92% of the detected mutation events were on A/C residues (Supplementary Figure S1D). Each condition was assayed in biological duplicate (roughly 2 months apart), yielding exceptionally high correlations (Figure 1C and Supplementary Figure S10A). Notably, while the *in vitro* and the denatured control samples slightly but significantly anticorrelated (average PCC = −0.07, Edgington-combined FDR-adjusted *P*-value = 3.3e–6), a moderate yet highly significant correlation was instead detected between the *in vivo* and the denatured control samples (average PCC = 0.18, Edgington-combined FDR-adjusted *P*-value < 2.2e–16). This suggests that, on average, IAV mRNAs are much less structured under *in vivo* conditions than they are *in vitro*. This was further confirmed by inspection of base reactivity distributions (Figure 1D), revealing how the median reactivity of IAV mRNAs *in vivo* (0.63) is closer to that of the denatured controls (0.69 and 0.68 respectively for the *in vitro* and *in vivo* denatured samples), than to that of the *in vitro* refolded sample (0.49).

### Structure modeling of *in vivo* IAV mRNA structures

To chart accurate experimentally-informed RNA secondary structure models of IAV mRNAs we next sought to incorporate DMS reactivities as soft constraints to guide thermodynamics-based RNA structure inference software (see Materials and Methods). This approach has been extensively validated (42,50–52), and previously applied to successfully infer viral RNA structures under both *in vitro* and *ex virio* conditions (21,26,27,53,54).

Instrumental to a successful chemical probing-guided modeling of RNA structures is the identification of optimal folding parameters (*slope* and *intercept*) that are needed to convert base reactivities into pseudo-free energy contributions (42,50). In order to identify the optimal slope/intercept pairs under our experimental conditions, complementary DMS-MaPseq datasets were produced by probing *E. coli* rRNAs under either *ex vivo* deproteinized, *in vitro*, or denaturing conditions (Supplementary Figure S2A). Compared to traditional DMS-seq and DMS-MaPseq data analyses, we here introduced a two-step normalization process, by first independently normalizing each dataset by 90% Winsorizing, followed by the normalization of both the *in vitro* and *in vivo* datasets onto the denatured control sample. This approach allowed us to both smooth the effect of under and over-reactive residues, and to increase the signal-to-noise ratio by accounting for the maximum background probability of each residue to react with DMS (Figure 1E and Supplementary Figure S2B–D). As a proof of its effectiveness, this 2-step normalization approach had negligible effects on base-paired residues (90% Winsorizing median reactivity: 0.04; two-step normalization median reactivity: 0.05), while it strongly increased the signal on unpaired residues (90% Winsorizing median reactivity: 0.53; two-step normalization median reactivity: 0.9). Under these conditions, grid search found optimal slope/intercept value pairs to be respectively 2.8 kcal mol^−1^ and −0.4 kcal mol^−1^ for the *in vivo* dataset, and 2.6 and −1 kcal mol^−1^ for the *in vitro* dataset (Supplementary Figure S2E and F, see Materials and Methods).

**Figure 2.**
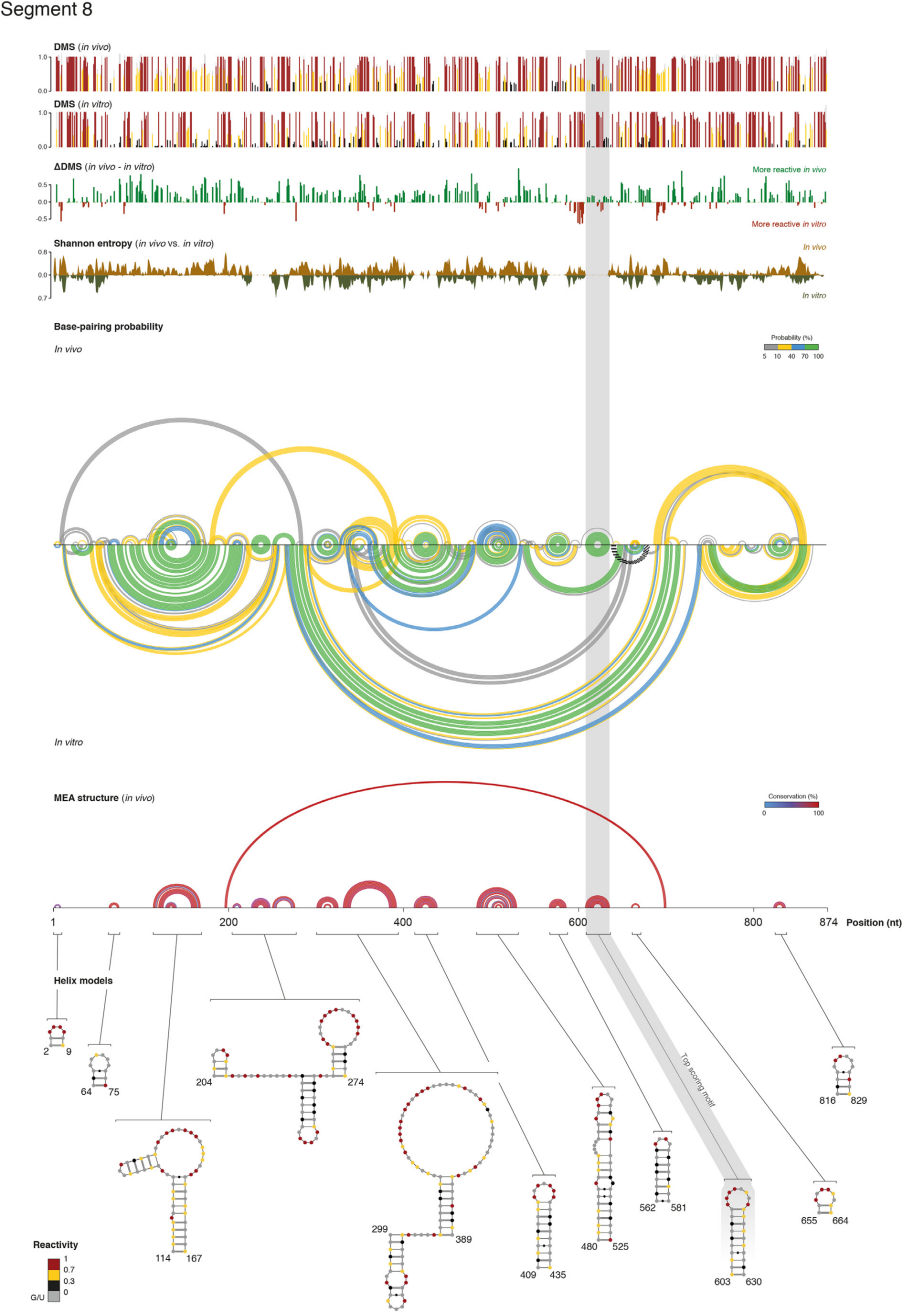
Transcriptome-wide *in vivo* secondary structure model of IAV mRNAs. *In vivo* and *in vitro* DMS reactivities, reactivity difference (ΔDMS), Shannon entropies, base-pairing probabilities, minimum expected accuracy (MEA) structure, and helix models with superimposed *in vivo* DMS reactivities for IAV segment 8, NS (NS1/NEP). Reactivity values are reported as the arithmetic mean of two biological replicates. Error bars represent SDs. Base-pairs are depicted as arcs, colored according to their probabilities. Green arcs correspond to base-pairs with *P* ≥ 0.7. Black dashed arcs correspond to pseudoknots. Regions with multiple overlapping arcs (high Shannon entropies) correspond to regions that are likely to form alternative structures. Base-pairs in the MEA structure are depicted as arcs, colored according to their conservation as determined by multi-sequence alignment of IAV mRNAs.

By incorporating the identified folding parameters into DMS-directed structure modeling, we here obtained the first high-quality single-base resolution secondary structure maps of the entire IAV transcriptome (nearly 13 500 nt, across 8 segments, 10 transcripts), under both *in vivo* and *in vitro* conditions (Figure 2 and Supplementary Figures S3–S18), with a median coverage of at least 6000× in each dataset. Besides performing minimum free energy (MFE) folding, partition function folding allowed us to calculate base-pairing probabilities and to infer per-base Shannon entropies. Shannon entropy can be used as a proxy to measure the likelihood of RNA regions to either form a single stable (low entropy) or multiple alternative (high entropy) structures (21).

### IAV mRNAs are largely unfolded *in vivo*, but retain crucial structured domains

Initial model-free inspection of our data through the Gini index (12,13) that quantifies the evenness of the distribution of DMS base reactivities, confirmed that IAV mRNAs are significantly less folded *in vivo* than *in vitro* (Supplementary Figure S19A). Accordingly, structure modeling confirmed that, on average, 80.3% of the IAV transcriptome is unfolded *in vivo* (Supplementary Figure S19B). This reduced propensity to fold into stable secondary structures is not due to sequence constraints leading to a limited folding space, as nearly half of the IAV transcriptome is instead stably folded *in vitro*, similarly to what is expected for structural RNAs (47.8% on average for mouse 18S/28S rRNAs). Rather, it is possibly the consequence of an active translation-mediated unfolding of RNA structures, as it has been recently reported for cellular mRNAs (12,24). Nonetheless, our analysis revealed the presence of a few persistent RNA structural motifs in IAV mRNAs *in vivo*. These structures mostly represent locally stable secondary structures, involving short-range base-pairs (average/median bp span: ∼49/17 nt). In contrast, larger structures form *in vitro*, often involving much longer base-pair interactions (average/median bp span: ∼75/31 nt, Supplementary Figure S19C). Roughly 55% of the base-pairs of the *in vivo* motifs were also predicted to occur *in vitro*. Conversely, only 23% of the base-pairs formed *in vitro* were vice versa present *in vivo* (Supplementary Figure S19D). The highest overall base-pairing conservation between *in vivo* and *in vitro* foldings was observed for segments 7 and 8 IAV mRNAs (respectively ∼67% and ∼75% of the base-pairs present *in vivo* were also present *in vitro*), while the lowest was observed for segment 4 IAV mRNA (only ∼32% of the base-pairs present *in vivo* were also present *in vitro*). These observations suggest that, while thermodynamics acts as a major driving force on the folding of segments 7 and 8 IAV mRNAs, other cellular factors might be involved in regulating the folding of segment 4 IAV mRNA. Pseudoknots were predicted for segments 1, 3, 4, 5 and 8 under *in vitro* conditions, while no pseudoknot was predicted to occur *in vivo*.

Knowledge of IAV RNA structure is very sparse, with most available data limited to *in vitro* probing and *in silico* predictions, particularly of segments 7 and 8 IAV mRNAs. Structural information is missing for most other segments (Supplementary Figure S20). By systematically analyzing all the 19 literature-available structure models, we found that respectively, 13 (68.4%) or 9 (∼47.3%) were supported at different extents either by our *in vitro* or *in vivo* data (Supplementary Figure S21). Most discrepancies can be easily explained by the fact that a large fraction of these structures was derived by *in vitro* probing of a small portion of the underlying IAV segment, outside of its natural context. In the context of the full segment, instead, they are usually part of larger domains, thus leading to completely different foldings *in vitro*, while they are mostly unfolded *in vivo*. Moreover, our structural data comes from probing of whole cells. We can’t rule out the possibility that certain structural elements involved in segments 7 and 8 splicing might form different structures between the nucleus and the cytoplasm.

We further exploited targeted DMS-MaPseq analysis to query, for the first time, the structure of the two short isoforms produced by the alternative splicing of segments 7 and 8 IAV mRNAs (Supplementary Figure S10), which yield the matrix 2 (M2) proton channel and nuclear export protein (NEP), respectively. These two isoforms are mostly unfolded compared to their full-length counterparts (Supplementary Figure S10B), but respectively retain 3 (nt 894–919, 950–964 and 967–994) and 2 (nt 562–581 and 603–630) hairpins of the full-length segments 7 and 8 (Supplementary Figure S10C and D). Interestingly, splicing of segment 7 results in the formation of an additional hairpin (nt 51–75 of the splice isoform) crossing the splice junction.

### Loss of conserved thermodynamically-favored structural motifs potently affects IAV fitness

**Figure 3.**
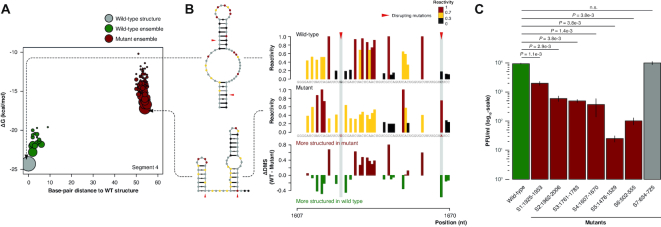
Targeted disruption of top-ranking IAV mRNA structural motifs potently affects viral fitness. (**A**) *In silico* design of a structure-disrupting mutant for IAV segment 4 (HA). Each circle corresponds to a different structure within the Boltzmann ensemble. The diameter of each circle corresponds to the log_2_ of the relative abundance of the respective structure within the ensemble. Free energies at 37°C and base-pair distances were computed in the absence of any experimental constraint; thus, the predicted structure might slightly differ from the experimentally-determined structure. (**B**) Targeted DMS-MaPseq analysis of the mutated top-ranking RNA structural motif for segment 4, HA (nt 1607–1670). 90% Winsorizing-normalized reactivities are reported and superimposed on both the WT and on the expected mutant structures. (**C**) IAV multi-cycle growth kinetics. MDCK cell monolayers were infected with WT or mutant IAVs at a MOI of 10^−4^. At 14 h post-infection, supernatants were harvested and viral titers determined by plaque assay. *P*-values are given by Welch's *t*-test statistics. Error bars correspond to SEs from three biological replicates.

To investigate whether the identified structural motifs have a biological role in the context of IAV replication, we next performed targeted mutagenesis aimed at disrupting these structures without affecting the underlying amino acid sequence. To prioritize the candidate structures most likely to be functional, we devised an empirical scoring function that weights more larger motifs (longer helices), with a higher probability of occurring under both *in vivo* and *in vitro* conditions (low Shannon entropies). Structures that are present both *in vivo* and *in vitro* are likely to be strongly thermodynamically favored. Furthermore, although sequence variation between IAV strains is too low to allow structure inference by covariation analysis (as confirmed by the analysis of multiple aligned IAV strain mRNA sequences using a recently published method aimed at statistically quantifying the evidence for structure conservation (55), *data not shown*), we reasoned that including base-pairing conservation in our score would at least penalize those RNA structural motifs that were not under strong purifying selection. Evaluating the conservation of IAV mRNA helices on a multiple sequence alignment of all available IAV subtypes (Supplementary Table S3), or restricting the analysis only to the H1N1 and H5N1 subtypes (Supplementary Table S4), only marginally altered the ranking of helices, but did not alter the top-ranking motifs. The top-ranking structural motif from each segment was then further selected for functional validation (highlighted in grey boxes in Figure 2 and Supplementary Figures S3–S9). However, we omitted the top-ranking structure from segment 8 and the splicing junction-crossing structural element in the short isoform of segment 7 IAV mRNA from our analysis, due to the difficulties connected with designing structure-disrupting mutations without affecting the underlying amino acid sequence of both encoded proteins. Mutants were designed by changing a maximum of three codons concurrently, without altering the encoded amino acid sequence and avoiding the introduction of rare codons. The mutant which maximized the predicted base-pair distance from the WT structure and minimized the frequency of the WT structure in the Boltzmann ensemble was selected (Figure 3A and Supplementary Figure S22). WT and mutant viruses bearing mutations that lead to disruption of IAV mRNAs 1 to 7 secondary structures were produced using a previously reported reverse genetics approach (49), and efficient structural disruption of the target motif was monitored by targeted DMS-MaPseq analysis (Figure 3B and Supplementary Figure S23). Although the extent of the observed structural alterations for the designed mutants was unpredictable solely from *in silico* prediction, as it ranged from a simple structural switch (e.g. segment 4), to complete unfolding of the target motif (e.g. segment 1), we were able to experimentally confirm the efficient disruption of all selected motifs. Strikingly, multi-cycle growth kinetics of WT and mutant viruses showed significantly attenuated replication kinetics at 14 h post-infection for mutated segments 1 to 6, ranging from 4 to 223-fold compared to WT IAV (Figure 3C). Surprisingly, we did not observe any significant effect for segment 7 mutant. Even though it is possible that this specific structural domain might not be essential for the efficient replication of IAV, a deeper inspection of DMS reactivities for this mutant revealed that the majority of bases undergoing a structural switch were shifting towards intermediate reactivity values, coherently with the presence of alternative RNA conformations. Hence, we can speculate that the introduced mutations might not be sufficient to completely abrogate the WT structure from the ensemble, thus resulting only in a moderate destabilization.

### Loss of segment 4 (HA) mRNA top-ranking structural motif is under strong negative selection

**Figure 4.**
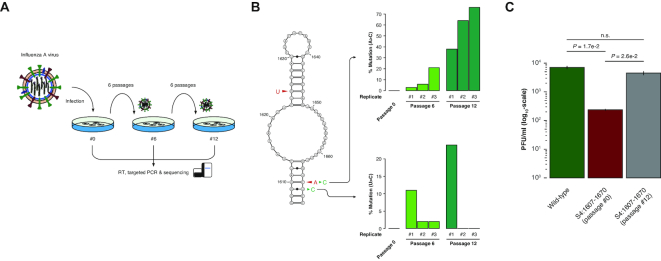
Spontaneous reversion of segment 4 mutant IAV. (**A**) Schematic of the *in vitro* IAV evolution experiment. (**B**) Selected structural motif for segment 4. Bases that were altered by targeted mutagenesis are indicated in red. Spontaneous mutations appearing during the time course of *in vitro* evolution are indicated in green. (**C**) IAV multi-cycle growth kinetics. MDCK cell monolayers were infected with WT or mutant 4 IAVs (either from passage 0 or 12 of the *in vitro* evolution experiment, replicate #1) at a MOI of 10^−4^. At 14 h post-infection, supernatants were harvested and viral titers determined by plaque assay. *P*-values are given by Welch's *t*-test statistics. Error bars correspond to SEs from 3 biological replicates.

We further hypothesized that, if the targeted disruption of these domains significantly affects the structural integrity of the IAV transcriptome, then a strong negative selection will be observed after several passages of infection, given the high mutation rate of RNA viruses (56). To this end, MDCK cells were infected with either WT or mutant IAVs, and the supernatant from each culture was used to repeat infection every 24 h, for a total of 12 passages of *in vitro* evolution (Figure 4A). We collected total RNA at passage 0, 6 and 12 of the time course experiment and performed targeted RT and sequencing of the mutated domains. After just six passages we observed the appearance of two independent mutations (A1667C and U1668C) within the motif from segment 4 (Figure 4B). These mutants are expected to revert the mutant motif to a WT-like structure by stabilizing the bottom stem, one by restoring the base-pair between positions 1610–1667 that we previously abolished by targeted mutagenesis, and the other by transforming the G:U wobble pair between positions 1609–1668 into a canonical GC pair, hence increasing the motif's stability by >2 kcal mol^−1^. This acquired U1668C mutation results in a change in the encoded amino acid from a Serine to a Proline. Notably, multiple sequence alignment of all IAV subtypes revealed that nearly any amino acid can occur at this position, although with different frequencies, and that a Proline naturally occurs in at least 2 IAV strains (Influenza A virus (A/goose/Guangdong/G3143/2014(H6)) and Influenza A virus (A/swine/QC/7780/2009(H1N1))). Altogether, our analysis suggests that stabilizing this RNA structural motif has higher relevance for IAV fitness than preserving the underlying amino acid sequence. Accordingly, multi-cycle growth kinetics assay performed for passage 12 IAVs confirmed rescue of the WT phenotype for this spontaneous segment 4 revertant (Figure 4C). Nonetheless, reversion to the WT sequence (A1667C) appears to have a competitive advantage over the U1668C mutation, as demonstrated by the disappearance of the latter in replicates 2 and 3 already at passage 12, and in replicate 1 at passage 22 (*data not shown*).

## DISCUSSION

The importance of RNA structure as a regulatory key player in the complex cellular environment has experienced increasing appreciation during recent years. This entailed fast method development (e.g. SHAPE-seq (57), RING-MaP (22), SHAPE-MaP (21), DMS-MaPseq (18), LASER-seq (58)), hence providing important insights into the biological role of RNA structure (23–25). The first genome-wide RNA structure model was resolved for HIV-1 in 2009 (26) and recently, RNA-RNA interactions of Zika virus were mapped *in vivo* (20). Also IAV RNA secondary structure has been in the focus of attention (28–40). However, no *in vivo* data for IAV mRNAs were available to date and the only genome-wide *in vivo* structure model for any virus available was published only recently for Zika virus (20).

We here introduced RAPiD-MaPseq, a fast and reliable approach that enables the targeted querying of long and low abundance RNAs with high precision and accuracy. As COMRADES (20), RAPiD-MaPseq relies on the high affinity of biotin and streptavidin, although COMRADES exploits direct coupling of biotin to the probing agent whereas RAPiD-MaPseq uses biotinylated oligonucleotides to enrich specific RNAs following DMS treatment. This has the advantage of allowing also RNAs with very low abundance to be structurally interrogated, while the sequencing depth of those RNAs is usually too low to yield informative results in the context of a transcriptome-wide experiment. By applying RAPiD-MaPseq to living cells infected with IAV, we here define for the first time *in vivo*, as well as *in vitro*, IAV mRNA secondary structures. Although binding of NP packaging proteins to both vRNAs and cRNAs largely inhibits the formation of RNA secondary structures (59,60), we cannot completely rule out the possibility that some structural domains we have here described might protrude from the NP-cRNA complex; a possibility that is supported by recent works showing that vRNAs-bound NP proteins are non-uniformly distributed (6,61,62).

IAV mRNAs appear to be mostly unfolded in living cells, analogously to what has been previously shown for mammalian mRNAs (12), oppositely to the tight conformation the IAV transcriptome adopts under *in vitro* conditions. Importantly, this is reflected by the number of literature-available structures that are supported by our data (∼70% for *in vitro*, ∼50% for *in vivo*), as available structures were mostly determined under *in vitro* conditions. However, although IAV mRNAs are mostly unfolded *in vivo*, we here identified several new *in vivo* stably-folded IAV mRNA structural motifs. After ranking these motifs according to their occurrence probability under both *in vivo* and *in vitro* conditions and to their conservation, we selected the top-scoring structure for each IAV mRNA segment and validated its biological importance by disrupting them through targeted mutagenesis. A similar approach was used by (41) to assess the effect of disrupting three structural motifs in the context of splicing of segments 7 (M1/M2) and 8 (NS1/NEP). The work presented herein extends this to the whole IAV mRNA transcriptome and targets structural motifs that were discovered *de novo* in the context of IAV infection in living host cells. Importantly, only disruption of a segment 7 IAV mRNA structural motif did not have any impact on the fitness of the virus, whereas all other tested motifs resulted in a striking attenuation of IAV replication.

Concerning the biological role of the RNA structural motifs we have here identified, at least three scenarios can be hypothesized. First, these structural elements could influence half-lives of the respective mRNAs. Accordingly, it has been previously shown that stem-loop structures close to the 3′ end can increase transcript half-lives (63) and that, generally, transcript stability and the presence of RNA secondary structures are interrelated (64,65).

Second, these structures might be involved in translational regulation. RNA structure is known to promote and regulate ribosome pausing (65–67). This mechanism has been previously proposed to be necessary to ensure proper co-translational folding of certain protein domains in the context of HIV-1 protein synthesis (26). Furthermore, the different distribution of locally-stable RNA structural elements along different IAV mRNAs might indirectly regulate the proportion of synthesized viral proteins as a consequence of different translation efficiencies.

A third possibility is that these structures might serve as docking platforms for specific protein interactors, or as decoys to sequester factors of the host's antiviral response in a similar fashion to certain viral proteins (68).

Although the mortality rate of Influenza has substantially decreased during the last century, according to WHO, 1 out of 1000 people who contract flu are estimated to die. Thus, the control of Influenza infections still poses major challenges. The current antiviral arsenal against IAV is in fact limited to three classes of FDA-licensed drugs: matrix protein inhibitors, neuraminidase inhibitors (NAIs) and the recently approved cap-dependent endonuclease inhibitors. Among the first, amantadine and rimantadine prevent the viral uncoating in endosomes by interfering with M2 ion channel-mediated acidification. However, nearly all the circulating IAV H1N1 and H3N2 viruses have evolved resistance to these drugs (69). NAIs, such as oseltamivir and zanamivir, on the other hand, inhibit the release of IAV particles from infected cells, and currently represent the first-line therapy against influenza infections. Despite their efficacy, however, the number of circulating NAI-resistant viruses has greatly increased in the past few years (70). Lastly, baloxavir is a selective inhibitor of the cap-dependent endonuclease activity of the polymerase acidic protein PA, that halts the cap snatching and therefore prevents viral mRNA synthesis (71,72). However, resistance to baloxavir has been reported already during phase II and III clinical trials (71). Given these facts, the development of novel anti-influenza agents that are effective against antigenically different viruses is urgently needed.

Targeting conserved IAV mRNA structural features represents an intriguing new possibility for the development of small molecule drugs. Indeed, the interest in the field is rapidly growing and entailing increased research and method development for high-throughput screenings of small molecules targeting RNAs (73,74). Biologically important RNA structures are often more conserved than the underlying amino acid sequence. Thus, targeting highly conserved viral RNA structures might lead to design and development of antiviral molecules less prone to rapidly become ineffective.

The *in vivo* transcriptome-wide map of IAV mRNA secondary structures presented here offers a plethora of novel *in vivo*-relevant IAV mRNA structural motifs, thus providing the unique opportunity to screen for therapeutically-active small molecule binders, similarly to what has been recently proposed for the Hepatitis C virus (HCV) (74). Moreover, extensive differences revealed by our comparative analysis of IAV mRNA structures under *in vivo* and *in vitro* conditions underline the importance of *in vivo* RNA structure determination. RAPiD-MaPseq is a decisive method for studying any RNA virus in its native *in vivo* conformation, including viruses that pose serious threats to public health.

## DATA AVAILABILITY

RAPiD-MaPseq data has been deposited to the Gene Expression Omnibus (GEO) database, under the accession GSE122286. Additional processed files are available at: http://www.incarnatolab.com/datasets/IAV_Simon_2019.php.

## Supplementary Material

gkz318_Supplemental_Files
