# Supplementary material for: In vivo analysis of influenza A mRNA secondary structures identifies critical regulatory motifs
Source: Nucleic Acids Res. 2019 May 4;47(13):7003–17. doi: 10.1093/nar/gkz318 (PMC6648356; doi:10.1093/nar/gkz318)
Supplement: gkz318_Supplemental_Files [file gkz318_supplemental_files.zip › Supplementary Data.pdf]

Supplementary Data

for

In vivo analysis of influenza A mRNA secondary structures identifies critical regulatory motifs

Lisa Marie Simon, Edoardo Morandi, Anna Luganini, Giorgio Gribaudo, Luis Martinez-Sobrido, Douglas H. Turner, Salvatore Oliviero and Danny Incarnato

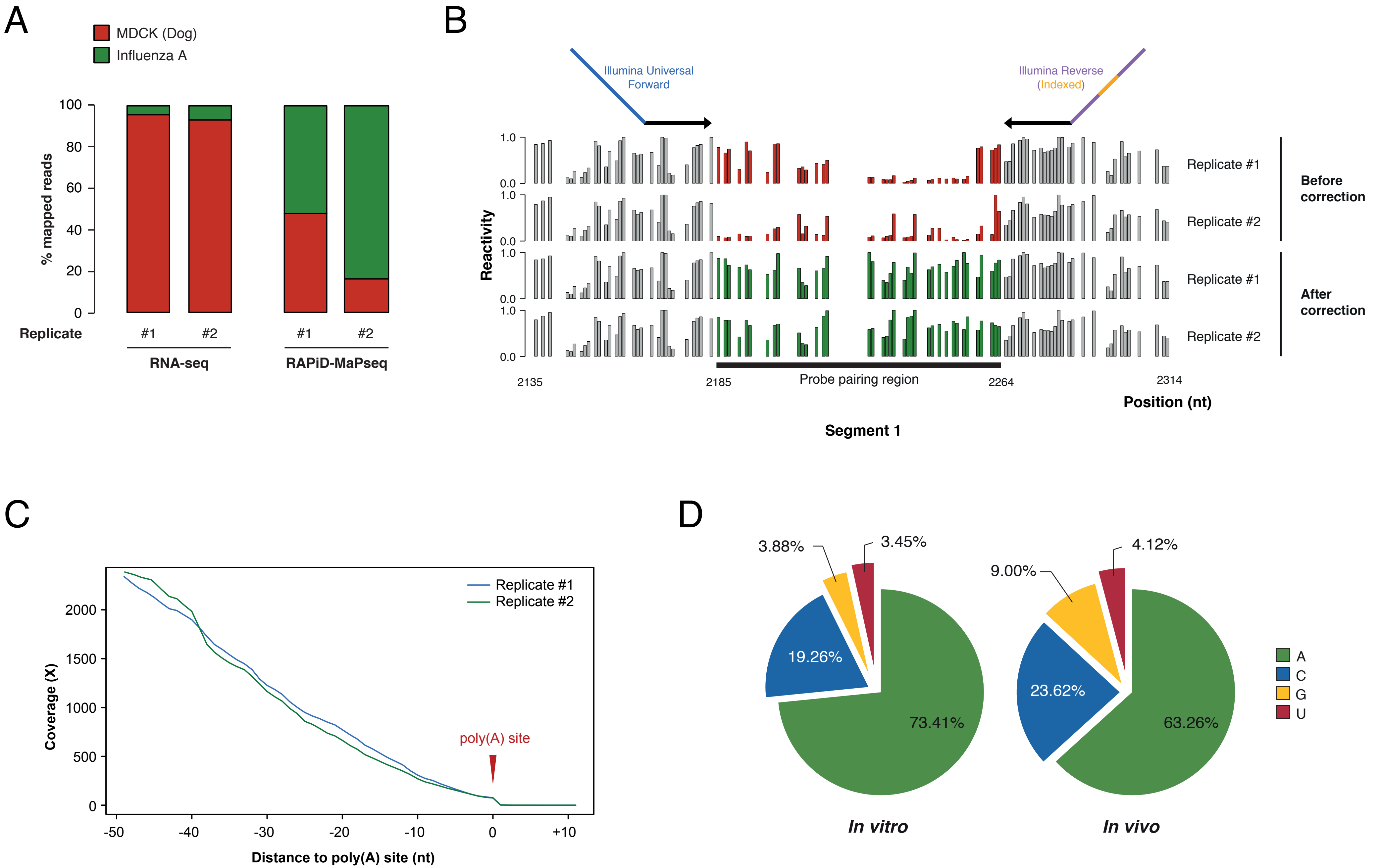

A
MDCK (Dog)
Influenza A
% mapped reads
100
80
60
40
20
0
Replicate
#1
#2
#1
#2
RNA-seq
RAPiD-MaPseq
B
Illumina Universal Forward
Illumina Reverse (Indexed)
Reactivity
1.0
0.0
Replicate #1
Replicate #2
Before correction
After correction
2135
2185
Probe pairing region
2264
2314
Position (nt)
Segment 1
C
Replicate #1
Replicate #2
Coverage (X)
2000
1500
1000
500
0
poly(A) site
-50
-40
-30
-20
-10
0
+10
Distance to poly(A) site (nt)
D
3.88%
3.45%
19.26%
73.41%
9.00%
4.12%
23.62%
63.26%
A
C
G
U
In vitro
In vivo

Figure S1. (**A**) Bar plot depicting the percentage of reads mapping on either dog genome (MDCK cells) or Influenza A mRNAs, before (RNA-seq) and after (RAPiD-MaPseq) RAP of IAV mRNAs. Multi-mapping reads were counted only if all their mapping positions univocally resided either on dog genome or Influenza A mRNAs. (**B**) Reactivity values on a 180 nt window centered on the pairing region of IAV segment 1 (PB2) RAPiD-MaPseq probe. Carryover of the DNA probe results in a low and variable structural signal on the region of probe annealing (red bars). Targeted DMS-MaPseq of this region enables the correction of the reactivity signal (green bars). (**C**) Mean coverage on the 3'-ends of IAV segments, aligned by their poly(A) sites, from the 2 *in vivo* RAPiD-MaPseq biological replicates, confirming efficient capture of IAV mRNAs only, as demonstrated by the drop of sequencing coverage exactly on poly(A) sites. (**D**) Pie charts showing the distribution of mutation frequencies on the four nucleotides for both the *in vitro* and *in vivo* DMS-MaPseq datasets. Data shown correspond to a single representative experiment.

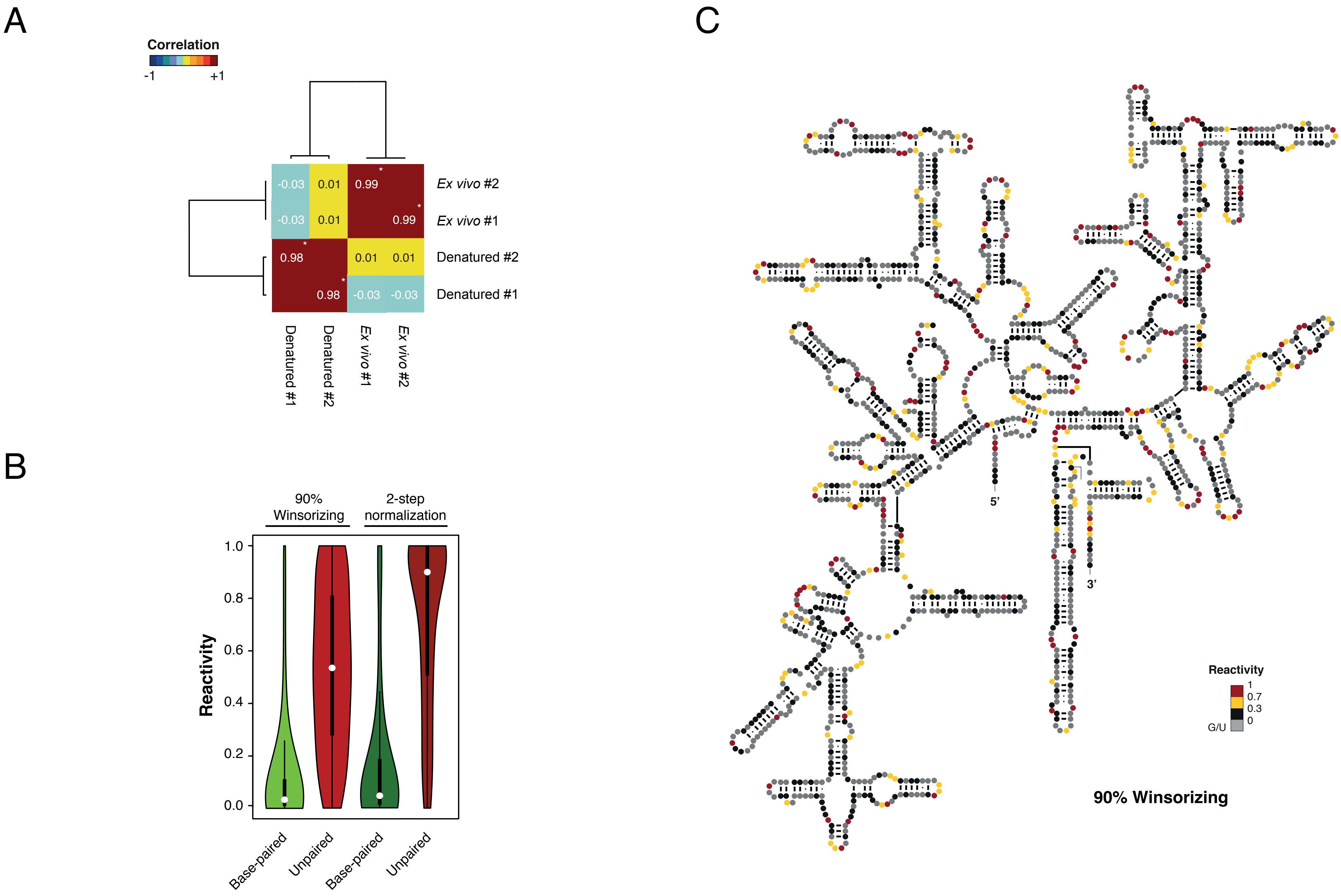
A
Correlation
-1
+1
-0.03
0.01
0.99
Ex vivo #2
-0.03
0.01
0.99
Ex vivo #1
0.98
0.01
0.01
Denatured #2
0.98
-0.03
-0.03
Denatured #1
Denatured #1
Denatured #2
Ex vivo #1
Ex vivo #2
B
90% Winsorizing
2-step normalization
Reactivity
1.0
0.8
0.6
0.4
0.2
0.0
Base-paired
Unpaired
Base-paired
Unpaired
C
5'
3'
Reactivity
1
0.7
0.3
0
G/U
90% Winsorizing

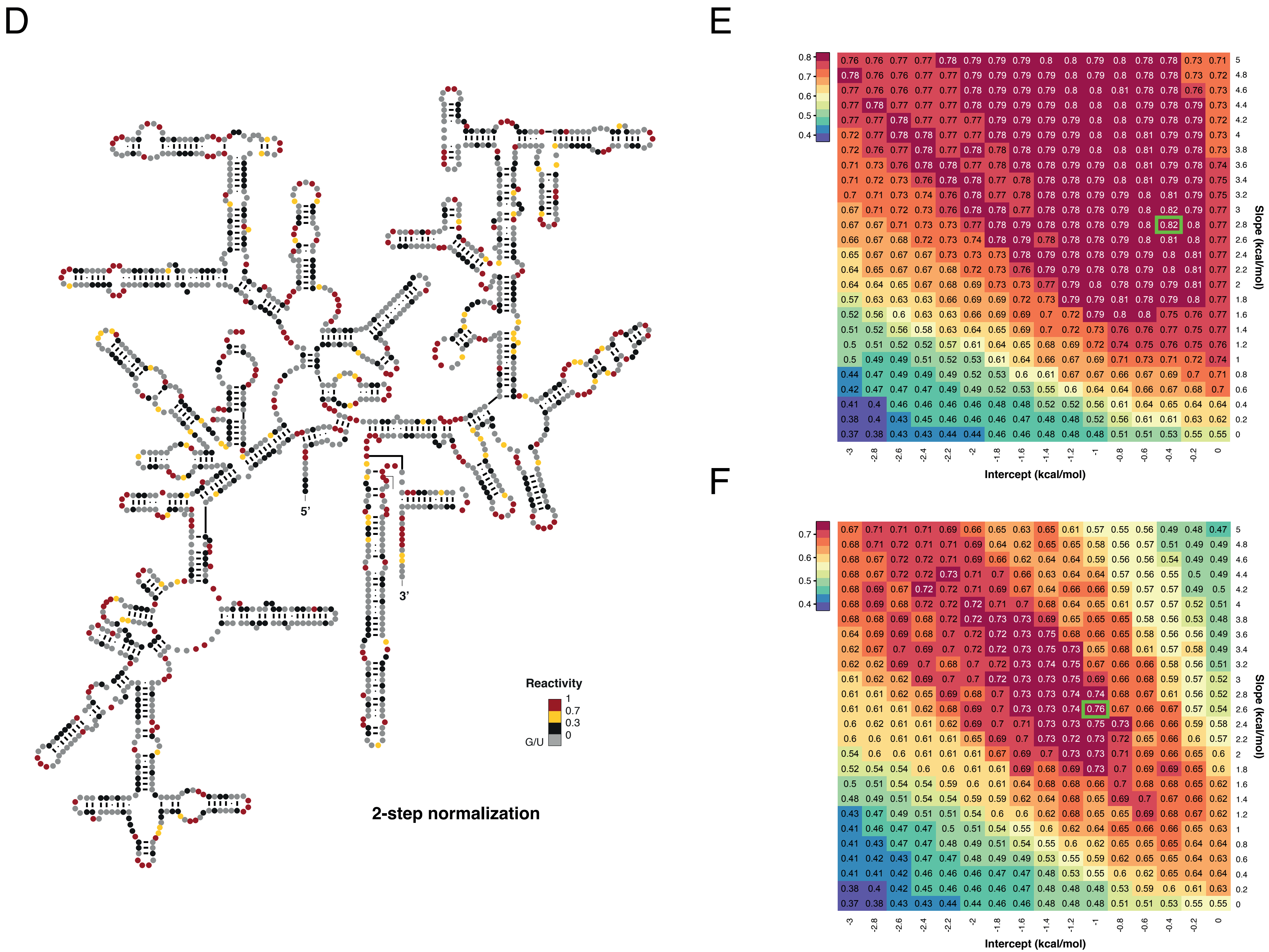
D
5'
3'
Reactivity
1
0.7
0.3
0
G/U
2-step normalization
E
Intercept (kcal/mol)
Slope (kcal/mol)
F
Intercept (kcal/mol)
Slope (kcal/mol)

Figure S2. (**A**) Heatmap of pairwise Pearson correlations for all *E. coli* DMS-MaPseq datasets. Significant correlations (p-value < 0.05) are denoted by a star in the upper-right corner. (**B**) Violin plot of reactivity value distributions on base-paired (green) and unpaired (red) residues for *E. coli* 16S/23S rRNAs either after 90% Winsorizing or 2-step normalization. (**C**) Phylogenetic structure of *E. coli* 16S rRNA with 90% Winsorizing-normalized reactivity values superimposed. The 16S structure model has been obtained from the CRW website (Cannone *et al.*, 2002). (**D**) Phylogenetic structure of *E. coli* 16S rRNA with 2-step normalized reactivity values superimposed. (**E**) Grid search (jackknifing) of optimal slope/intercept value pair for *in vivo* data. Values represent the geometric mean of sensitivity and PPV for the *E. coli* 16S/23S rRNA secondary structures predicted using each slope/intercept value pair. The chosen value pair is boxed in green. (**F**) Grid search of optimal slope/intercept value pair for *in vitro* data (as in **E**).

Segment 1

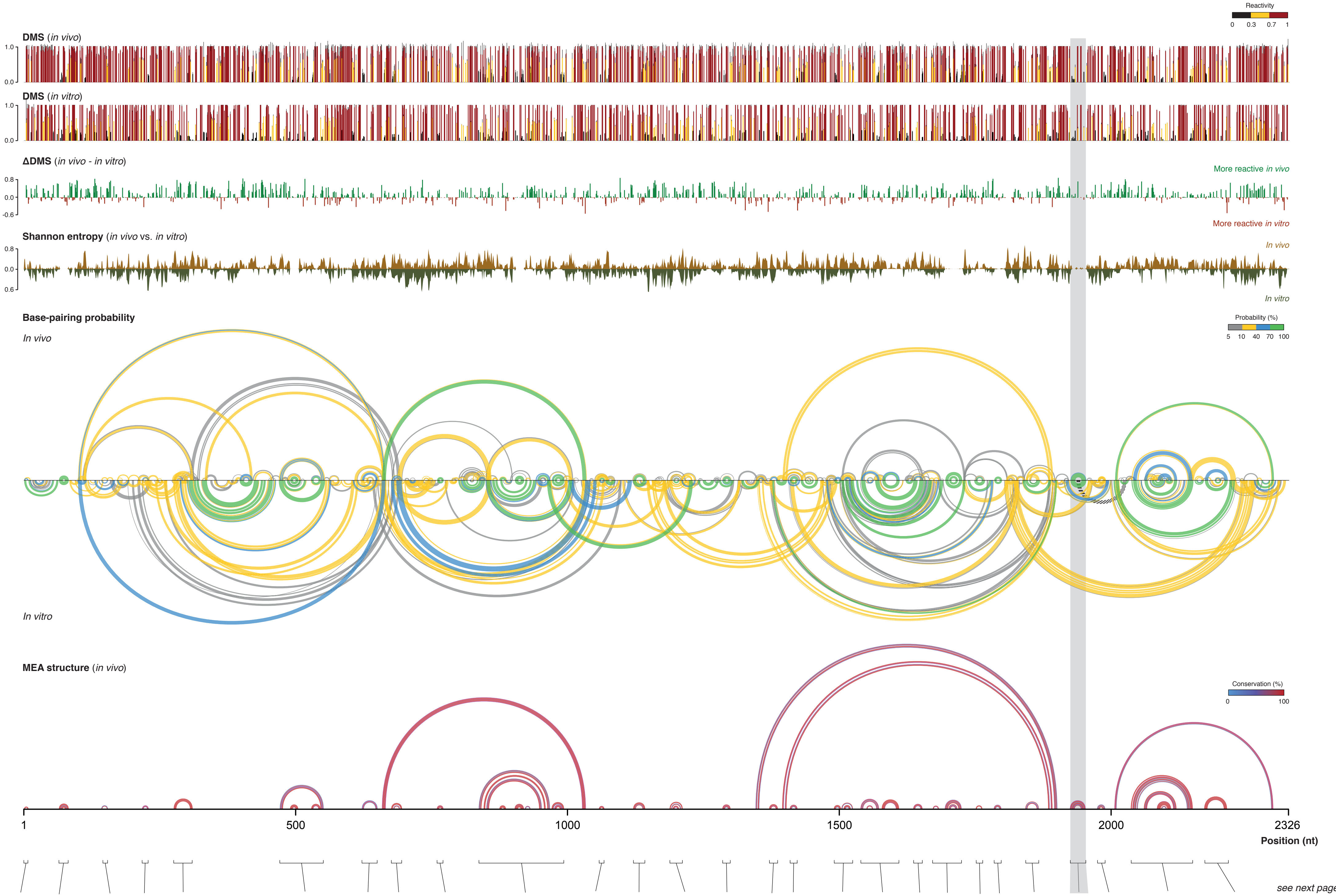

see next page

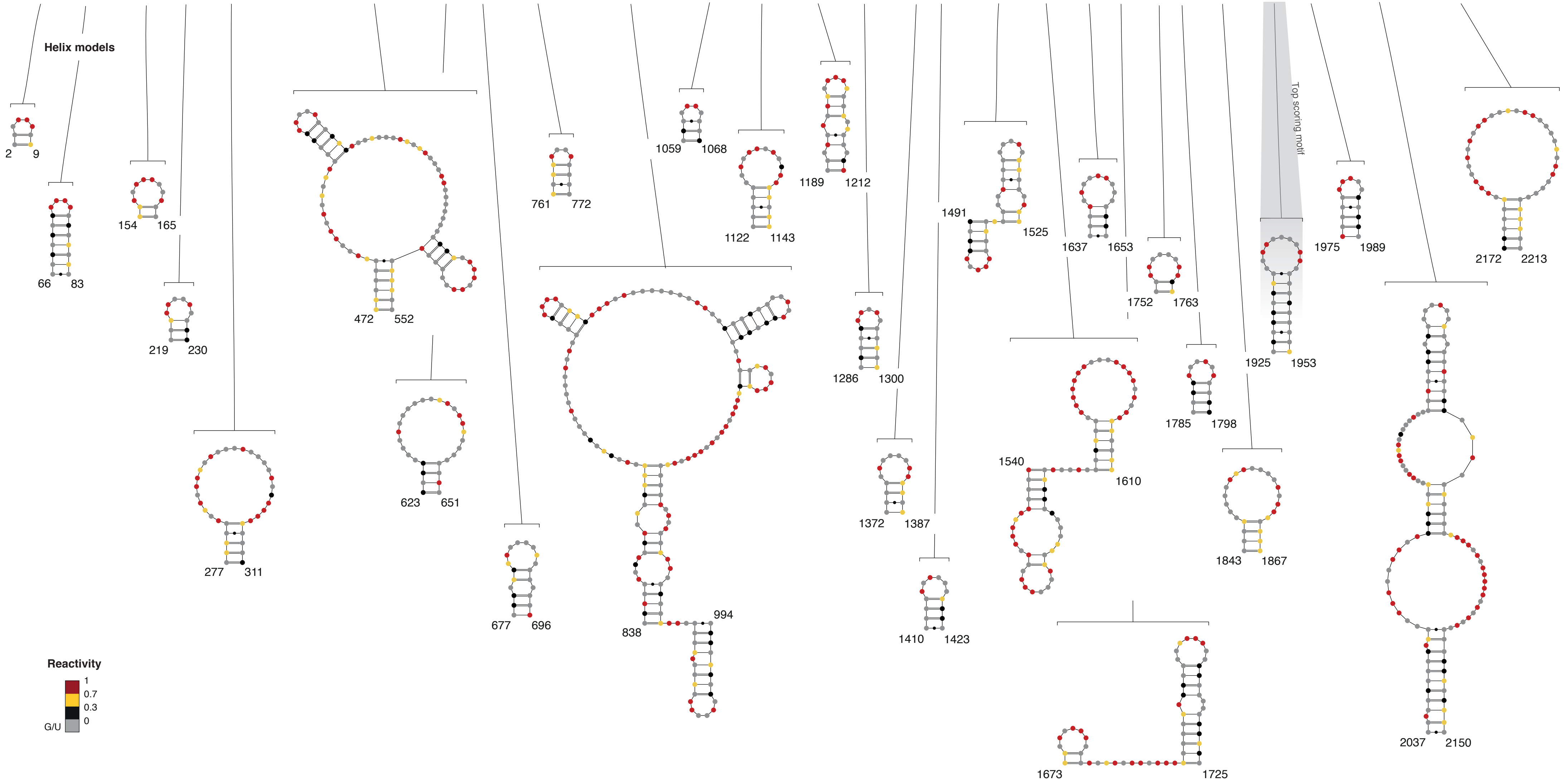

Helix models
2
9
66
83
154
165
219
230
277
311
472
552
623
651
677
696
761
772
838
994
1059
1068
1122
1143
1189
1212
1286
1300
1372
1387
1410
1423
1491
1525
1540
1610
1637
1653
1673
1725
1752
1763
1785
1798
1843
1867
1925
1953
Top scoring motif
1975
1989
2037
2150
2172
2213
Reactivity
1
0.7
0.3
0
G/U

Figure S3. *In vivo* secondary structure model for IAV segment 1 (PB2) mRNA. *In vivo* and *in vitro* DMS reactivities, reactivity difference (ΔDMS), Shannon entropies, base-pairing probabilities, minimum expected accuracy (MEA) structure, and helix models with superimposed *in vivo* DMS reactivities are reported. Reactivity values are reported as the arithmetic mean of 2 biological replicates. Error bars represent SDs. Base-pairs are depicted as arcs, colored according to their probabilities. Green arcs correspond to base-pairs with P ≥ 0.7. Black dashed arcs correspond to pseudoknots. Regions with multiple overlapping arcs (high Shannon entropies) correspond to regions that are likely to form alternative structures. Base-pairs in the MEA structure are depicted as arcs, colored according to their conservation as determined by multi-sequence alignment of IAV mRNAs.

Segment 2

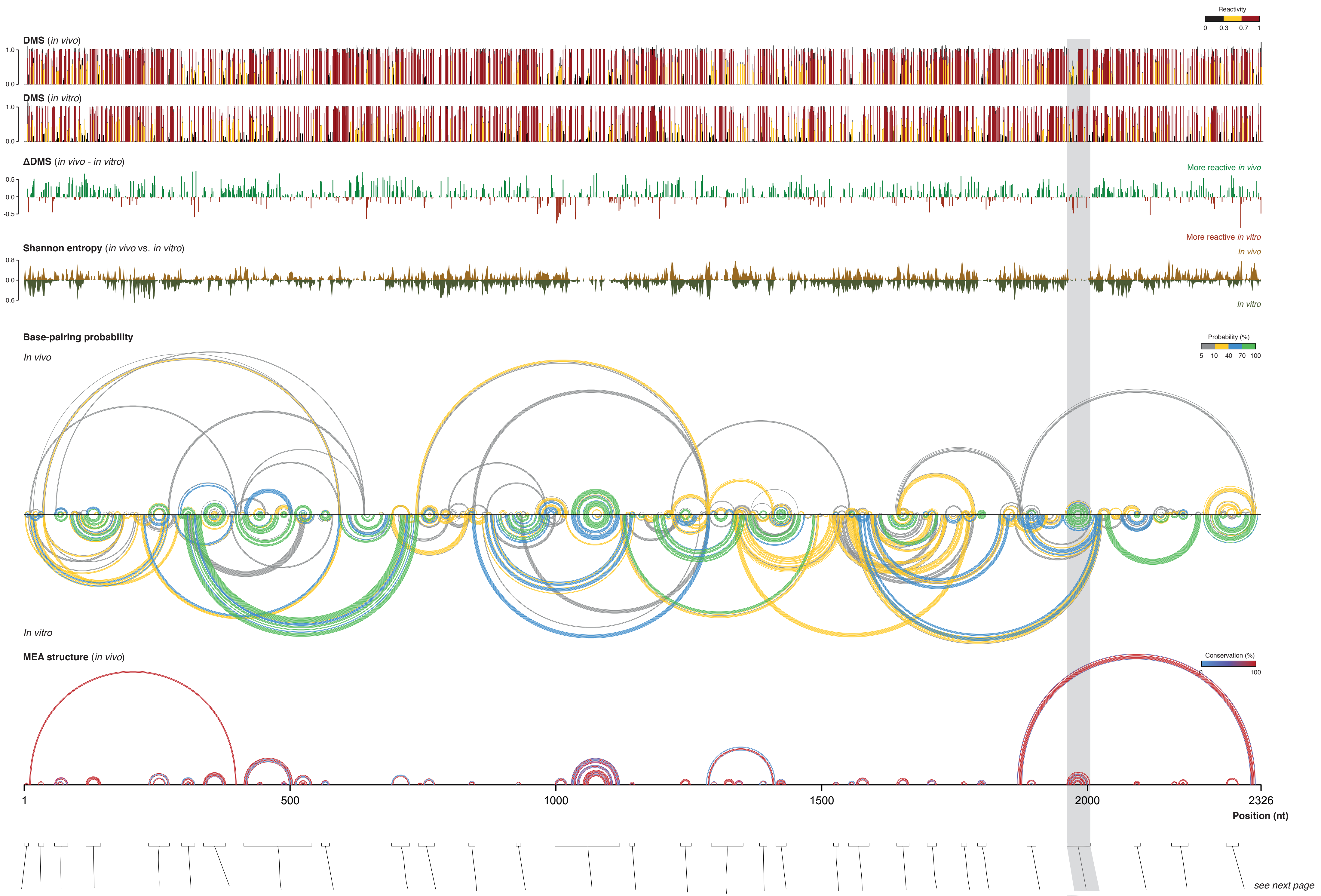

see next page

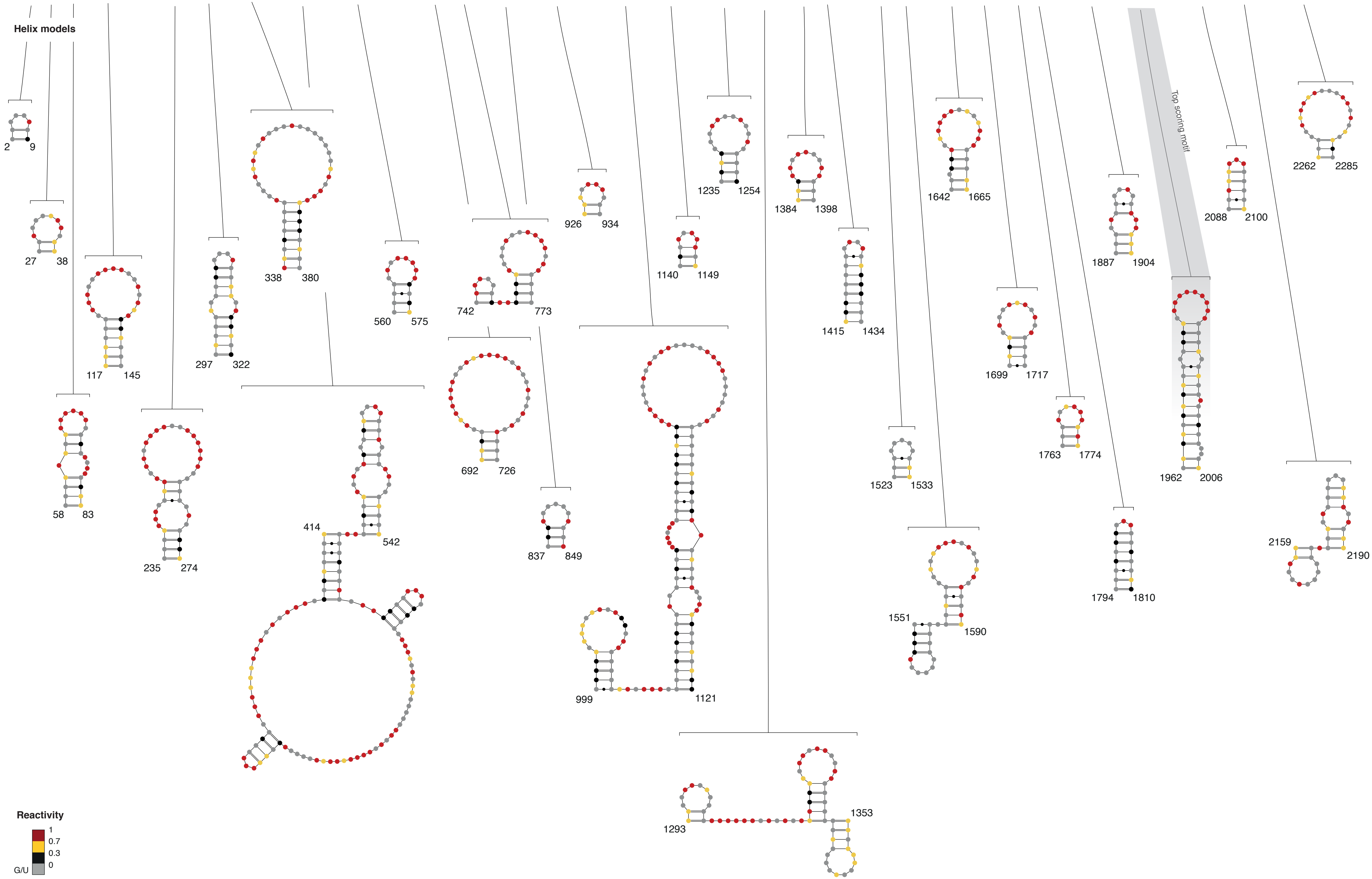

Helix models
2
9
27
38
58
83
117
145
235
274
297
322
338
380
414
542
560
575
692
726
742
773
837
849
926
934
999
1121
1140
1149
1235
1254
1293
1353
1384
1398
1415
1434
1523
1533
1551
1590
1642
1665
1699
1717
1763
1774
1794
1810
1887
1904
Top scoring motif
1962
2006
2088
2100
2159
2190
2262
2285
Reactivity
1
0.7
0.3
0
G/U

Figure S4. *In vivo* secondary structure model for IAV segment 2 (PB1) mRNA.

Segment 3

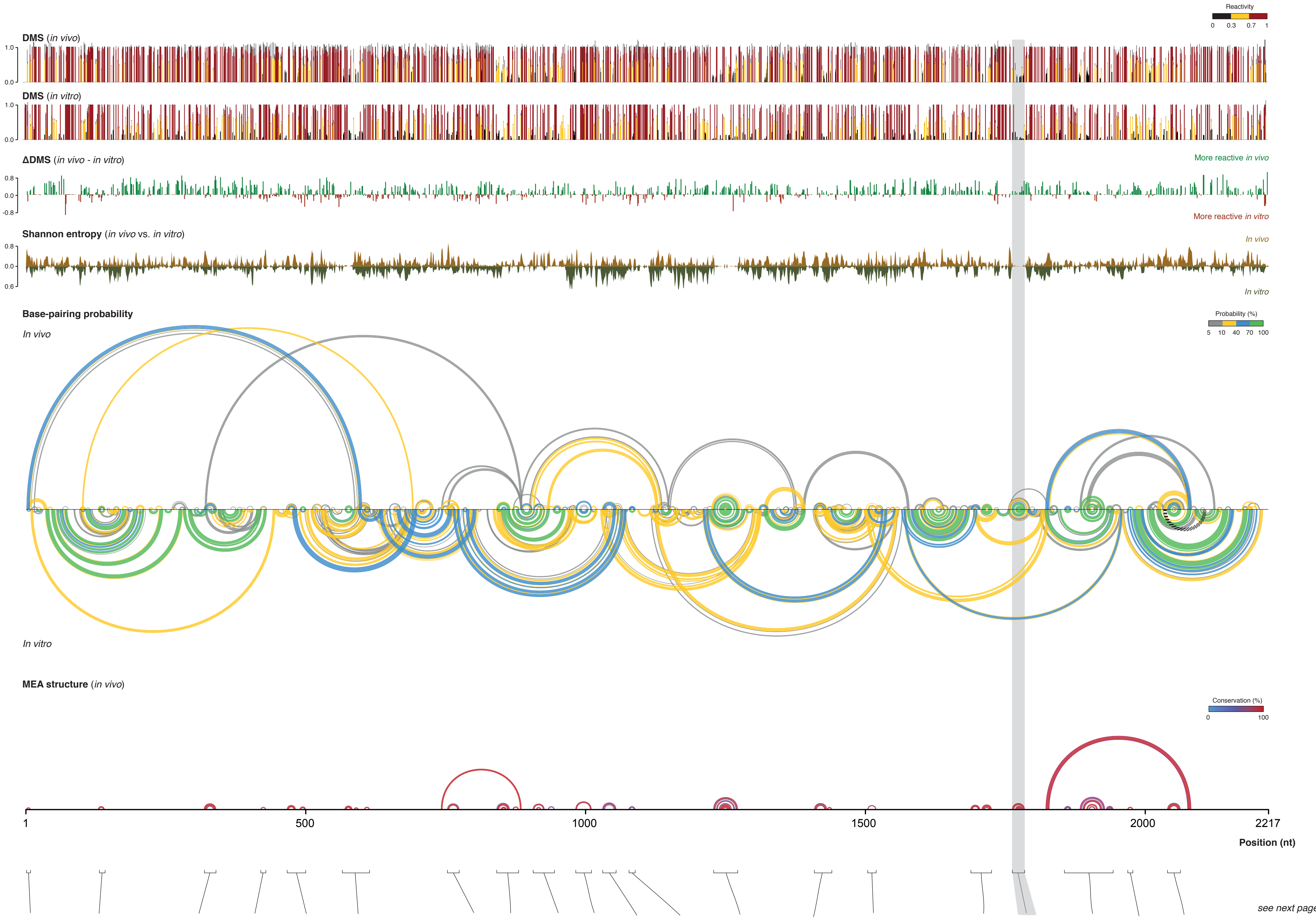

see next page

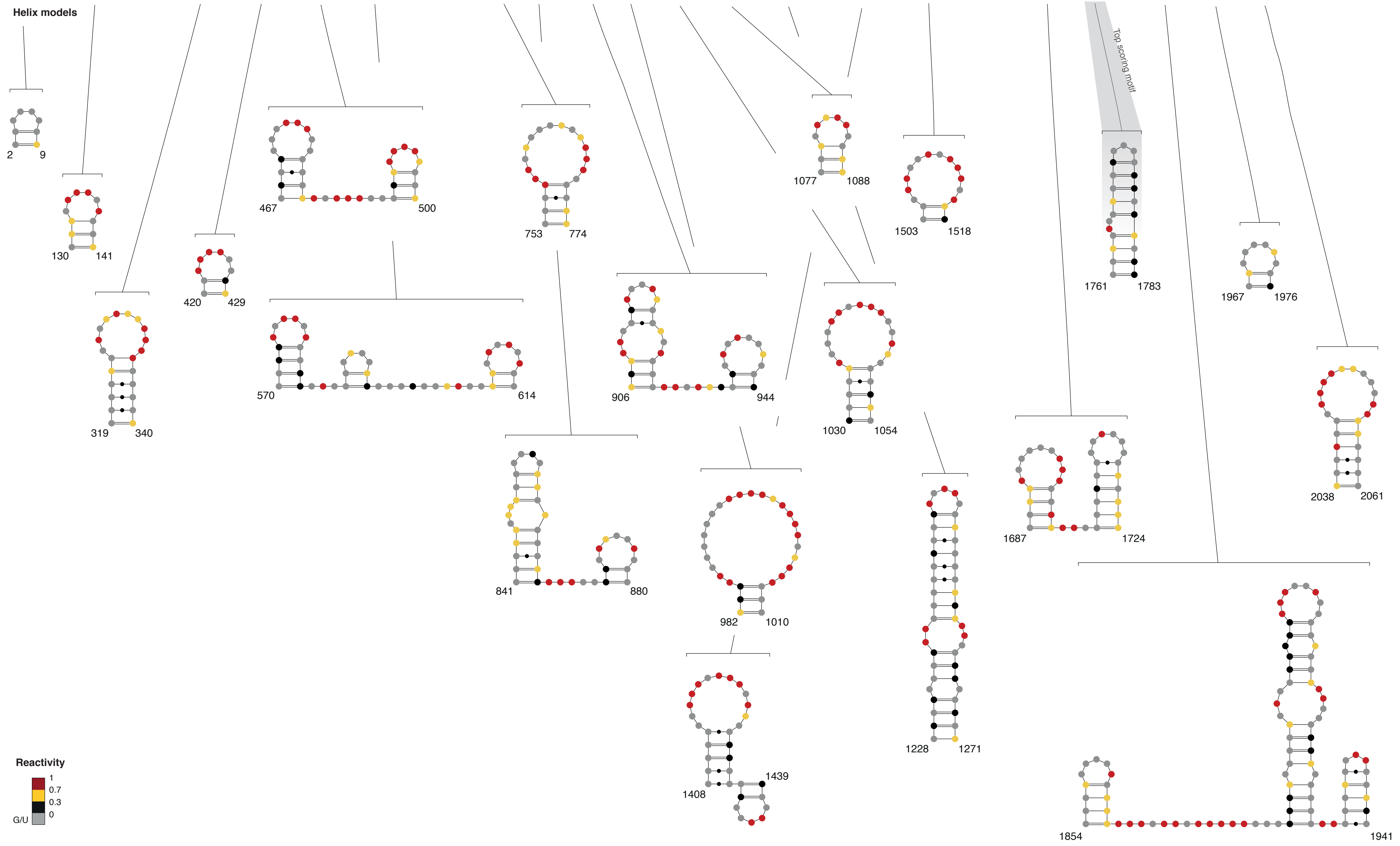

Helix models
Top scoring motif
2
9
130
141
319
340
420
429
467
500
570
614
753
774
841
880
906
944
982
1010
1030
1054
1077
1088
1228
1271
1408
1439
1503
1518
1687
1724
1761
1783
1854
1941
1967
1976
2038
2061
Reactivity
1
0.7
0.3
0
G/U

Figure S5. *In vivo* secondary structure model for IAV segment 3 (PA) mRNA.

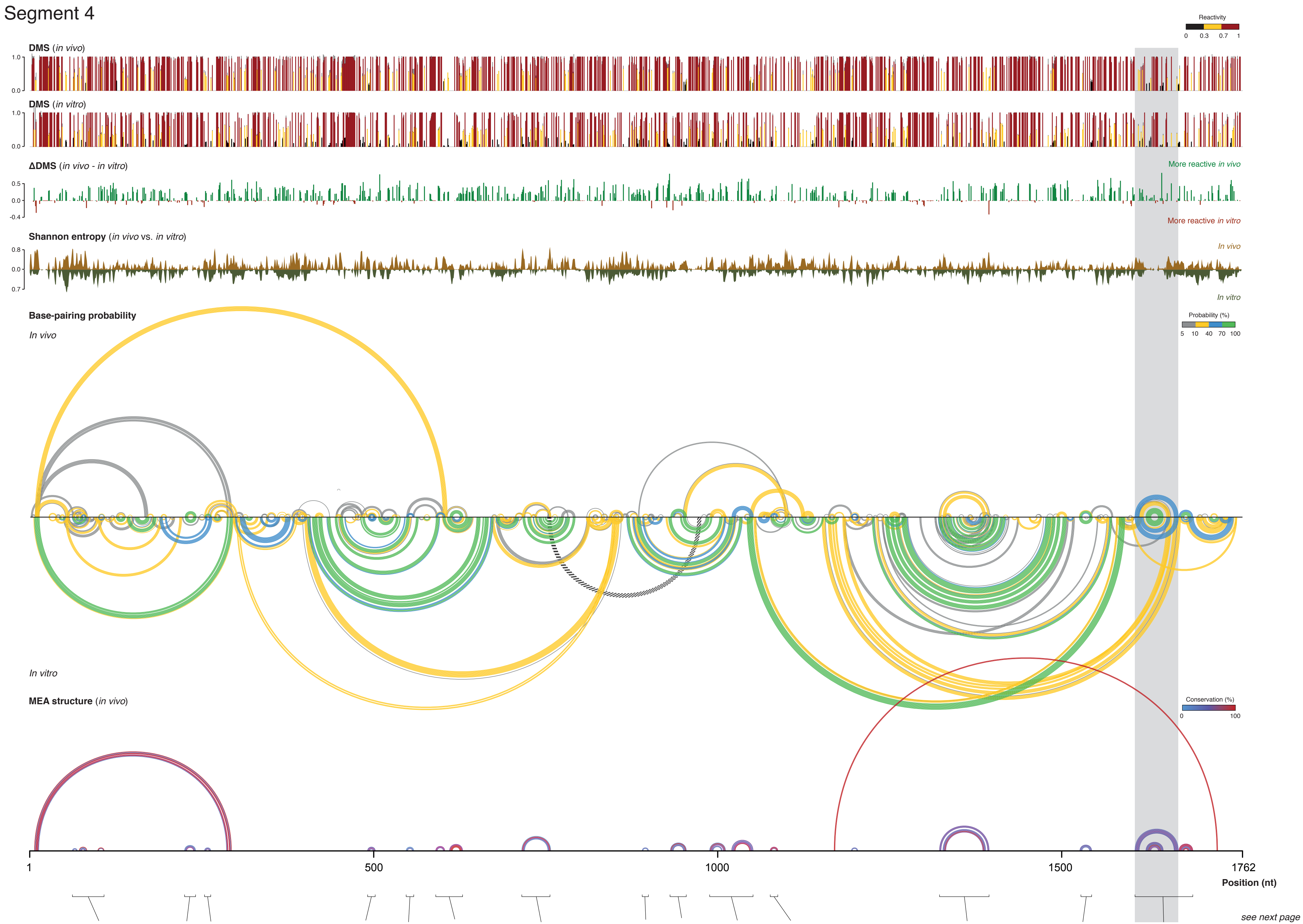

see next page

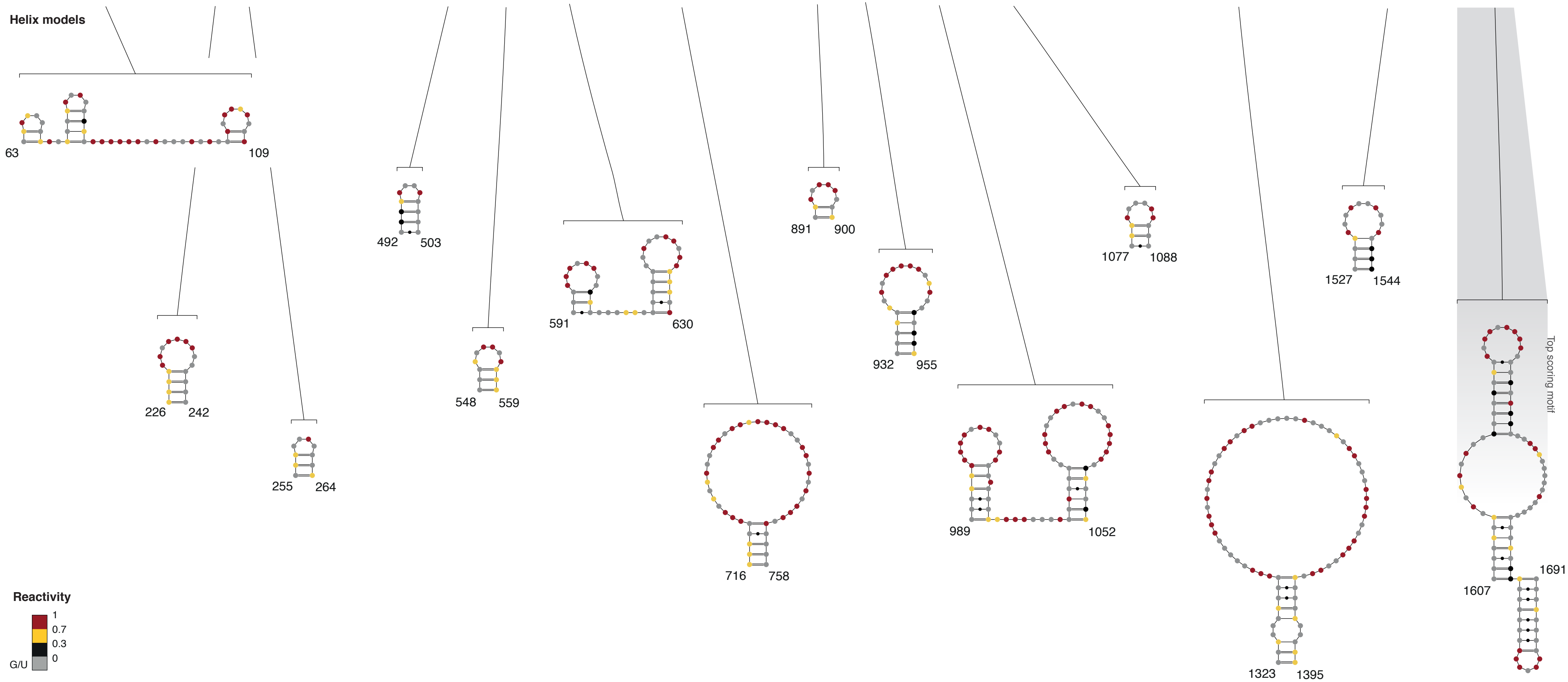
Helix models
63
109
226
242
255
264
492
503
548
559
591
630
716
758
891
900
932
955
989
1052
1077
1088
1323
1395
1527
1544
1607
1691
Top scoring motif
Reactivity
1
0.7
0.3
0
G/U

Figure S6. *In vivo* secondary structure model for IAV segment 4 (HA) mRNA.

Segment 5

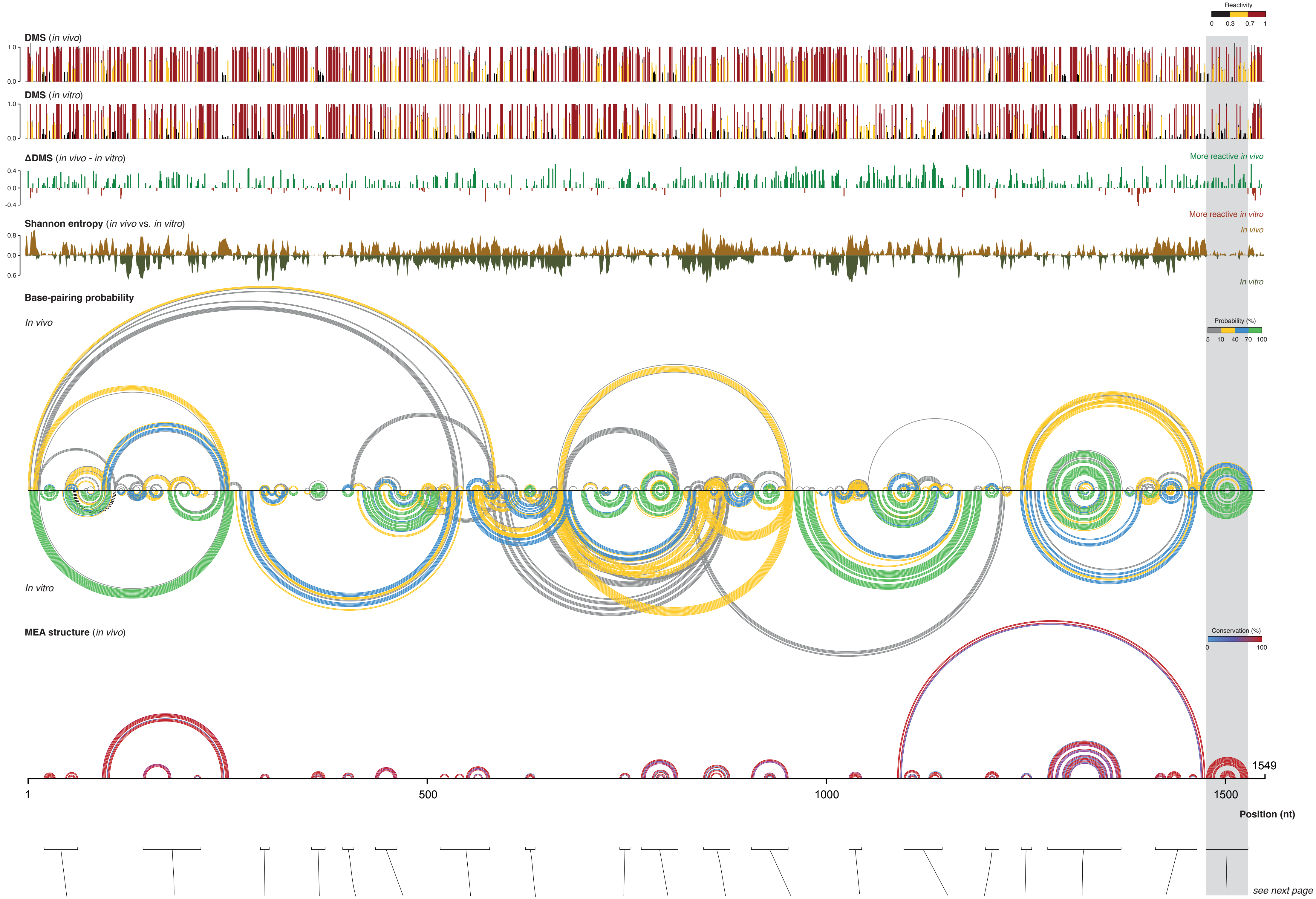

see next page

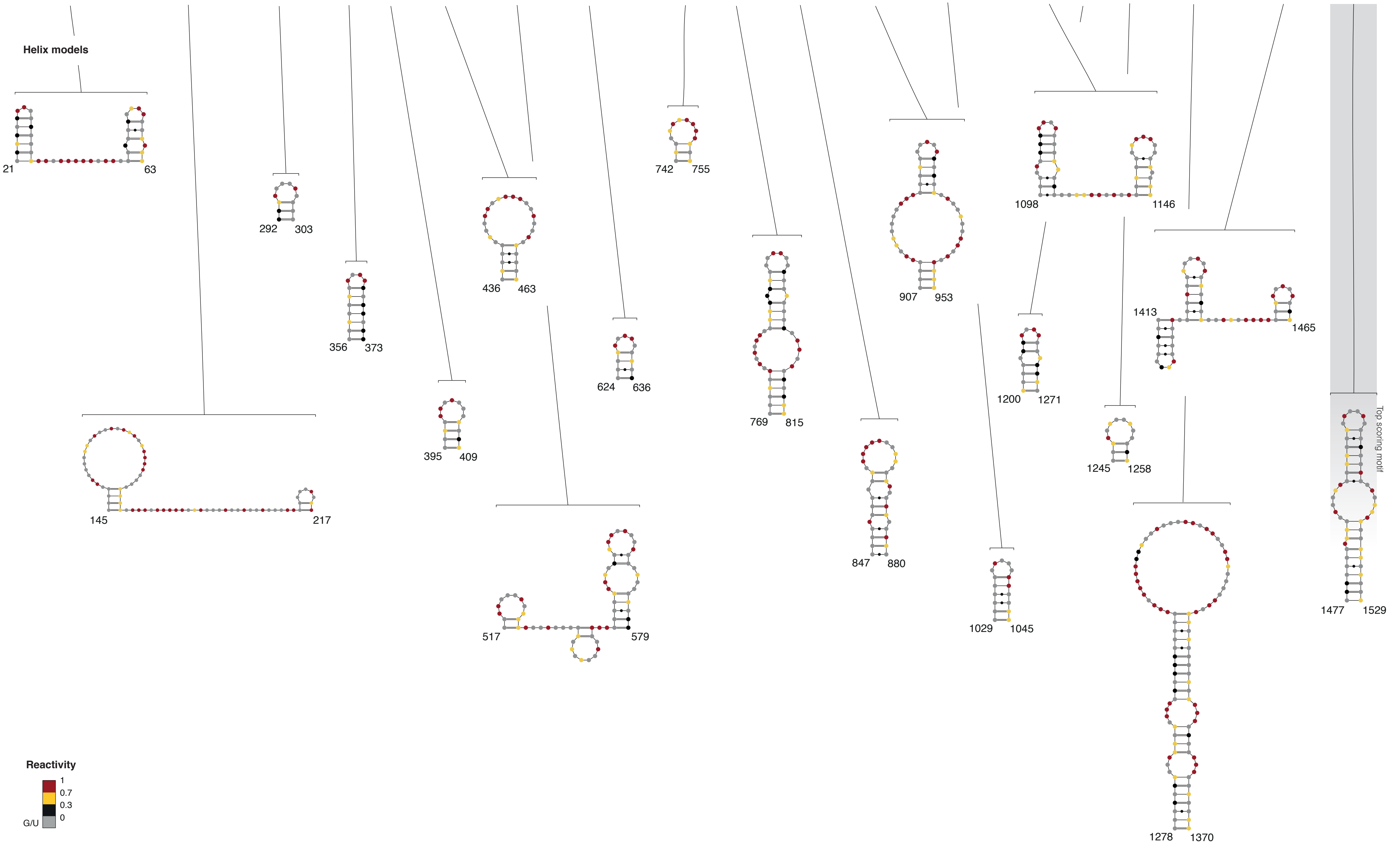

Helix models
21
63
292
303
356
373
395
409
436
463
517
579
624
636
742
755
769
815
847
880
907
953
1029
1045
1098
1146
1200
1271
1245
1258
1278
1370
1413
1465
1477
1529
145
217
Top scoring motif
Reactivity
1
0.7
0.3
0
G/U

Figure S7. *In vivo* secondary structure model for IAV segment 5 (NP) mRNA.

Segment 6

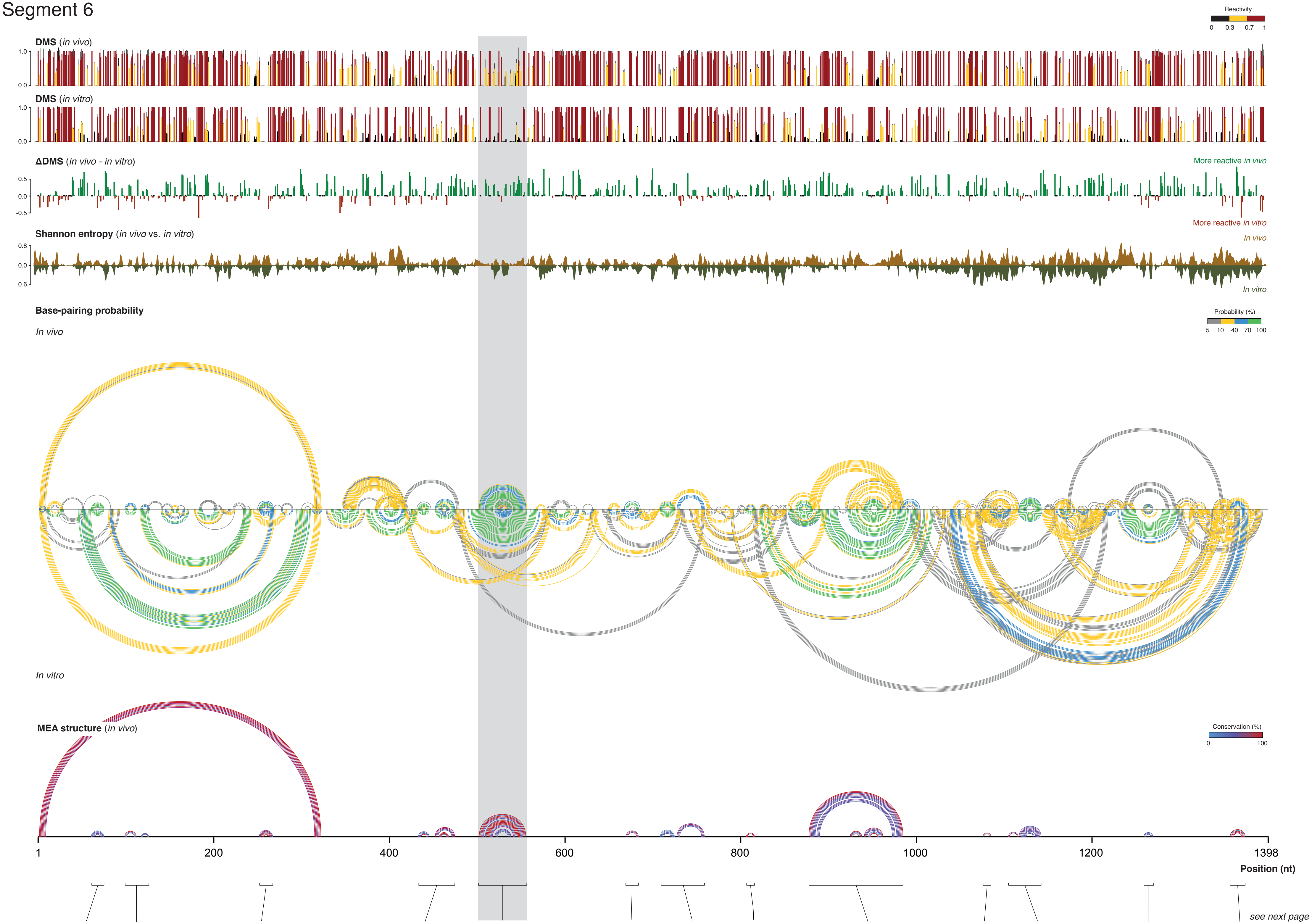

see next page

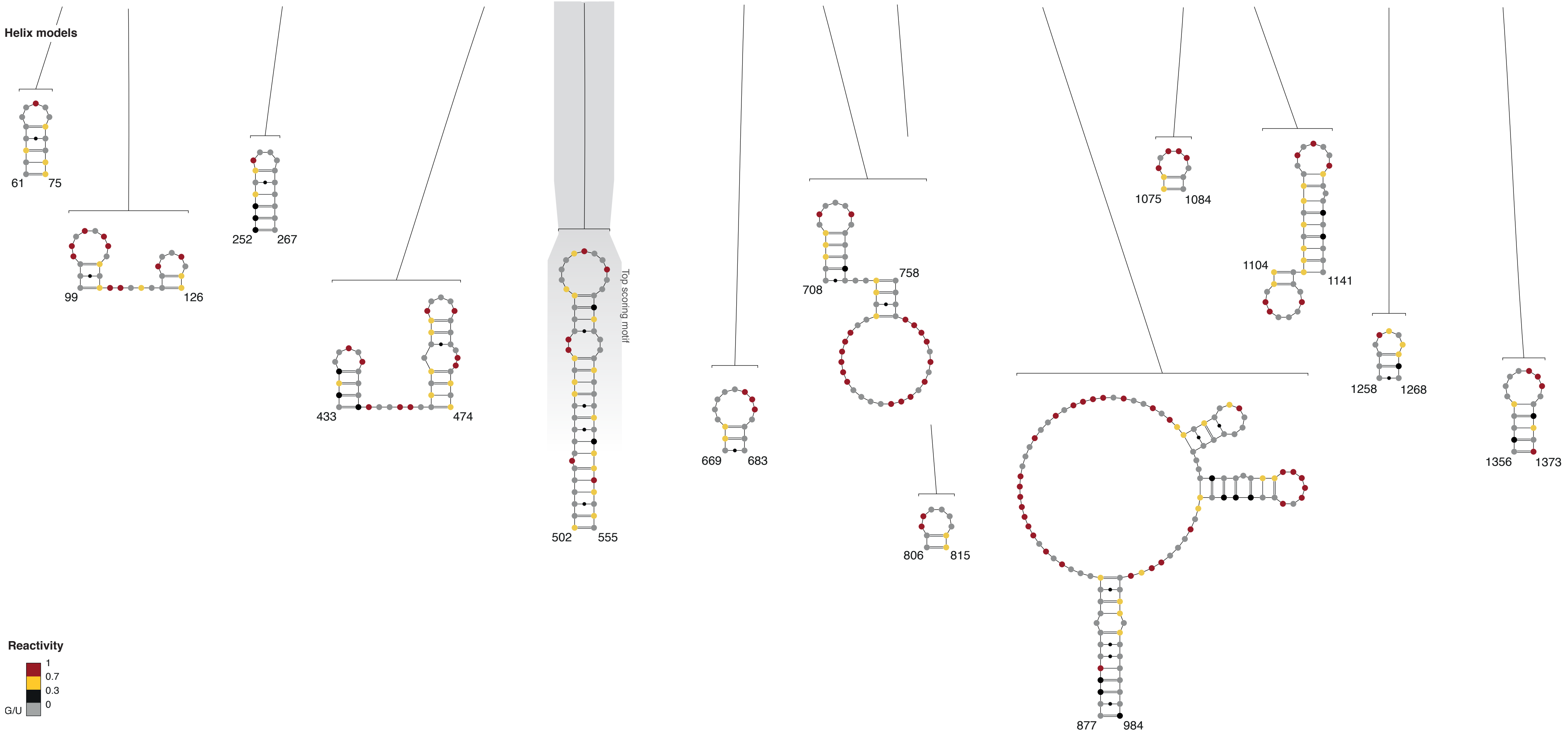

Helix models
61
75
99
126
252
267
433
474
Top scoring motif
502
555
669
683
708
758
806
815
877
984
1075
1084
1104
1141
1258
1268
1356
1373
Reactivity
1
0.7
0.3
0
G/U

Figure S8. *In vivo* secondary structure model for IAV segment 6 (NA) mRNA.

Segment 7

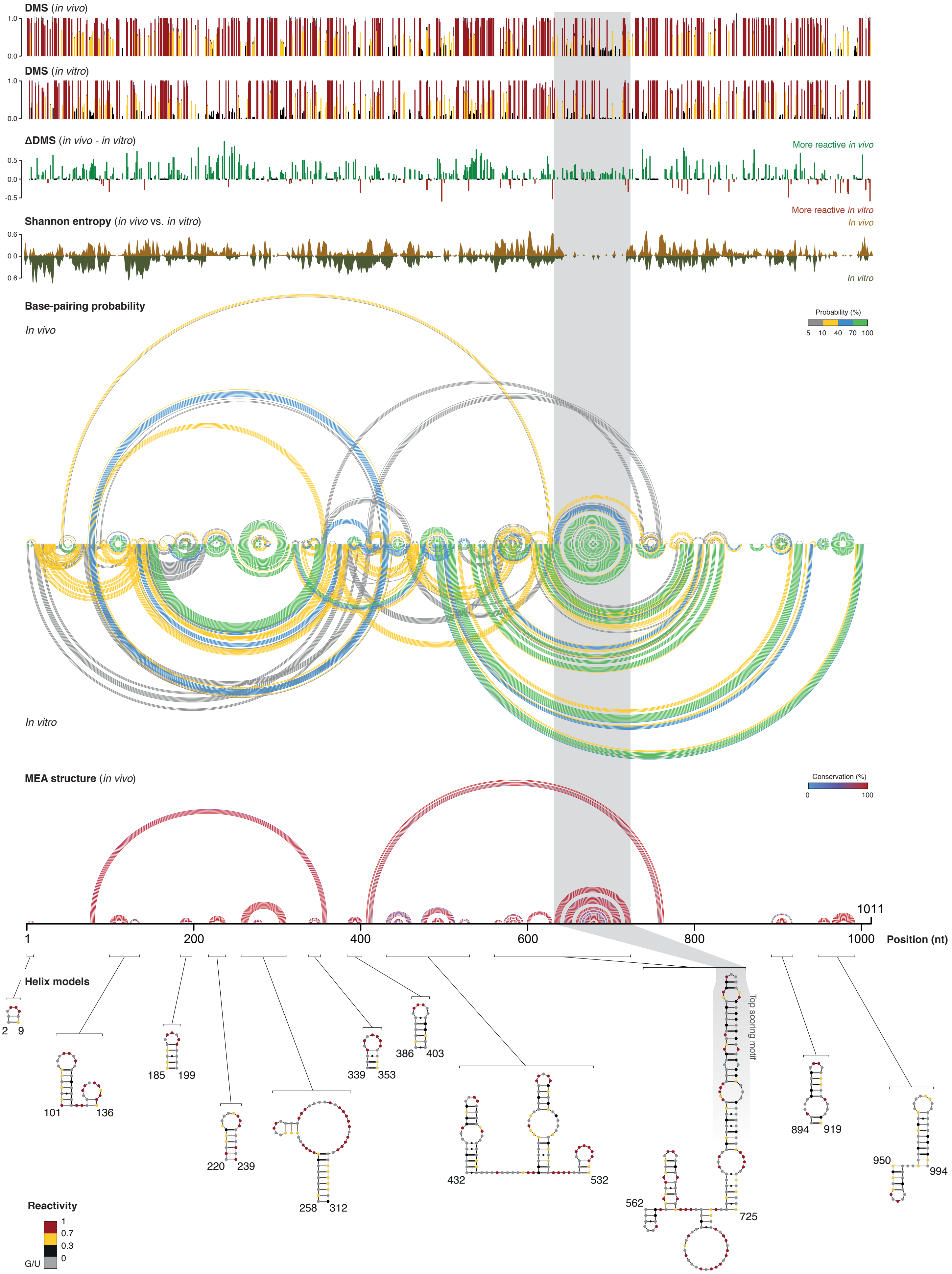

DMS (in vivo)
1.0
0.0
DMS (in vitro)
1.0
0.0
ΔDMS (in vivo - in vitro)
0.5
0.0
-0.5
More reactive in vivo
More reactive in vitro
Shannon entropy (in vivo vs. in vitro)
0.6
0.0
0.6
In vivo
In vitro
Base-pairing probability
In vivo
Probability (%)
5 10 40 70 100
In vitro
MEA structure (in vivo)
Conservation (%)
0 100
1011
1
200
400
600
800
1000
Position (nt)
Helix models
2 9
101 136
185 199
220 239
258 312
339 353
386 403
432 532
562 725
Top scoring motif
894 919
950 994
Reactivity
1
0.7
0.3
0
G/U

Figure S9. *In vivo* secondary structure model for IAV segment 7, M (M1/M2) mRNA.

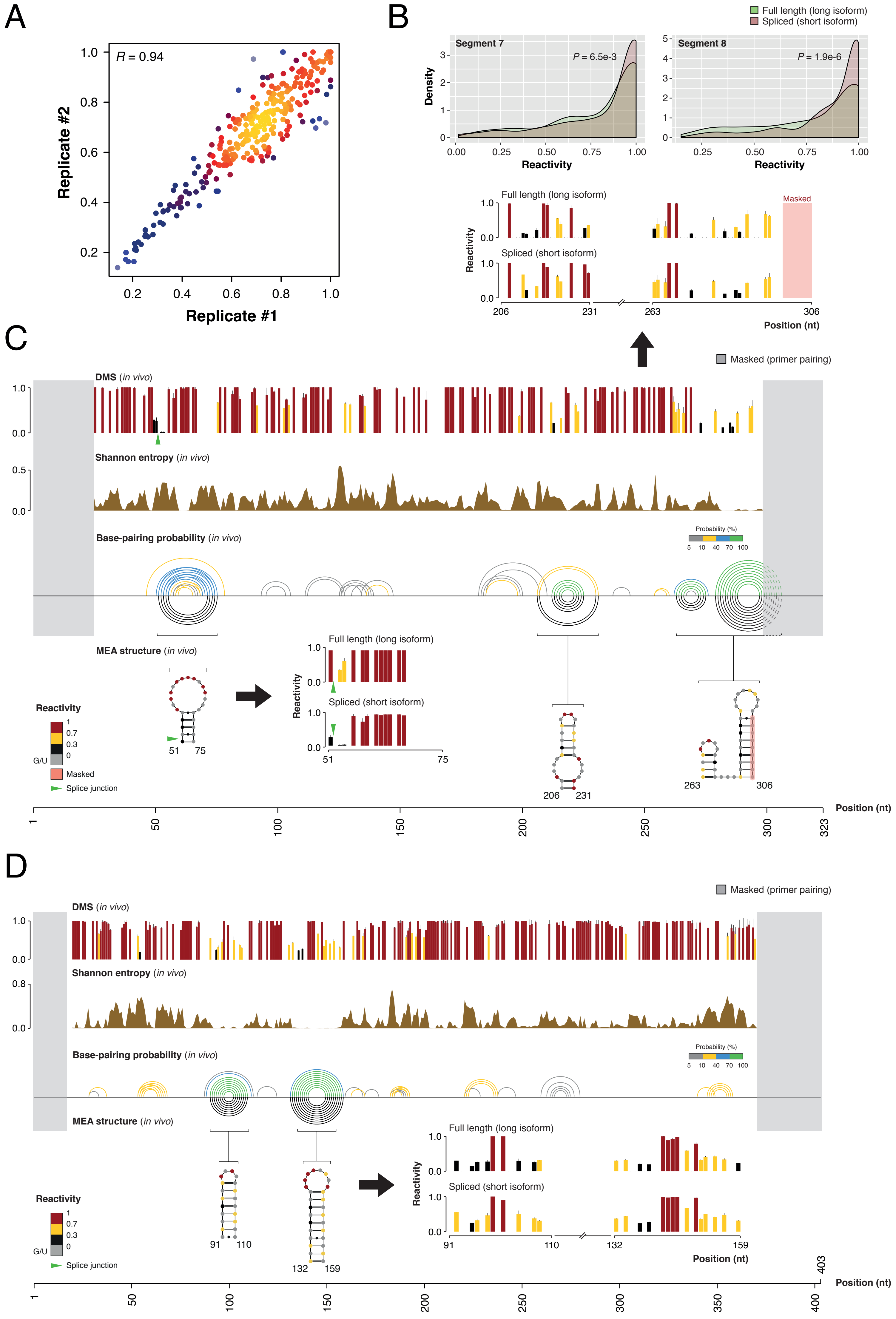

A
B
C
D
R = 0.94
Replicate #2
Replicate #1
Full length (long isoform)
Spliced (short isoform)
Segment 7
Segment 8
P = 6.5e-3
P = 1.9e-6
Density
Reactivity
Masked
Position (nt)
Masked (primer pairing)
DMS (in vivo)
Shannon entropy (in vivo)
Base-pairing probability (in vivo)
Probability (%)
MEA structure (in vivo)
Reactivity
G/U
Splice junction

Figure S10. (**A**) Heat-colored scatter plot of DMS reactivities for segment 7 (M) and 8 (NS) splice isoforms, across 2 biological replicates (PCC = 0.94). (**B**) Overlaid density plots of base reactivity distributions for both the full-length and the spliced isoforms of segments 7 and 8. P-values are given by paired Wilcoxon test statistics. (**C**) *In vivo* DMS reactivities, Shannon entropies, base-pairing probabilities, minimum expected accuracy (MEA) structure, and helix models with superimposed *in vivo* DMS reactivities for segment 7 short splice isoform (M2). Reactivity values are reported as the arithmetic mean of the 2 biological replicates. Error bars represent SDs. Base-pairs are depicted as arcs, colored according to their probabilities. Green arcs correspond to base-pairs with $P \geq 0.7$. Regions with multiple overlapping arcs (high Shannon entropies) correspond to regions that are likely to form alternative structures. For each structural motif in the MEA structure, the reactivity profile of the corresponding nucleotides in the segment's full-length mRNA is reported. Regions masked by primer binding, for which no structural information is available, are either marked by grey boxes or outlined in red on the secondary structure models. The splice junction is indicated by a green arrow. (**D**) *In vivo* DMS reactivities, Shannon entropies, base-pairing probabilities, minimum expected accuracy (MEA) structure, and helix models with superimposed *in vivo* DMS reactivities for segment 8 short splice isoform (NEP). Symbols and coloring are as in panel **C**.

Segment 1 (*in vitro*)

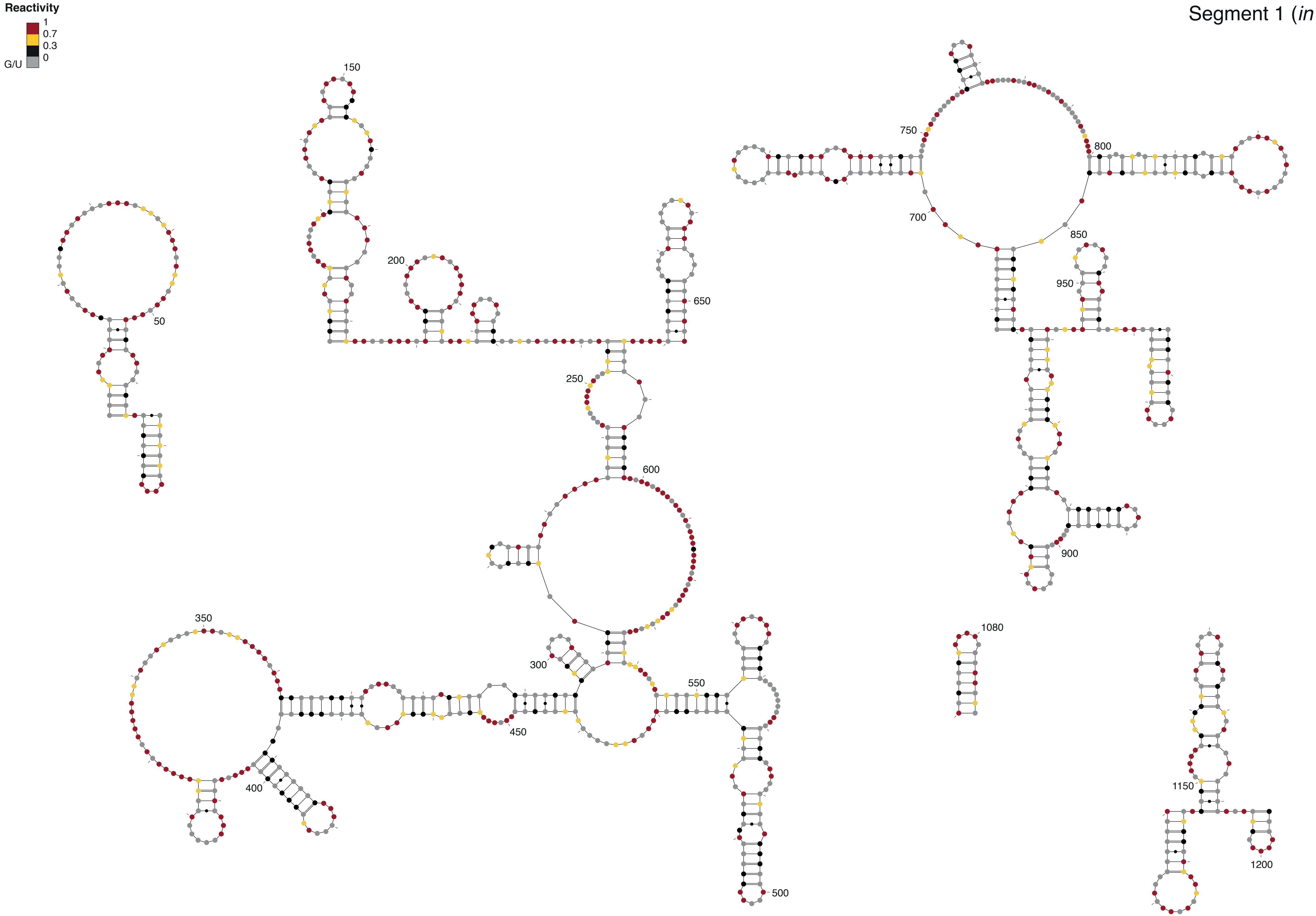

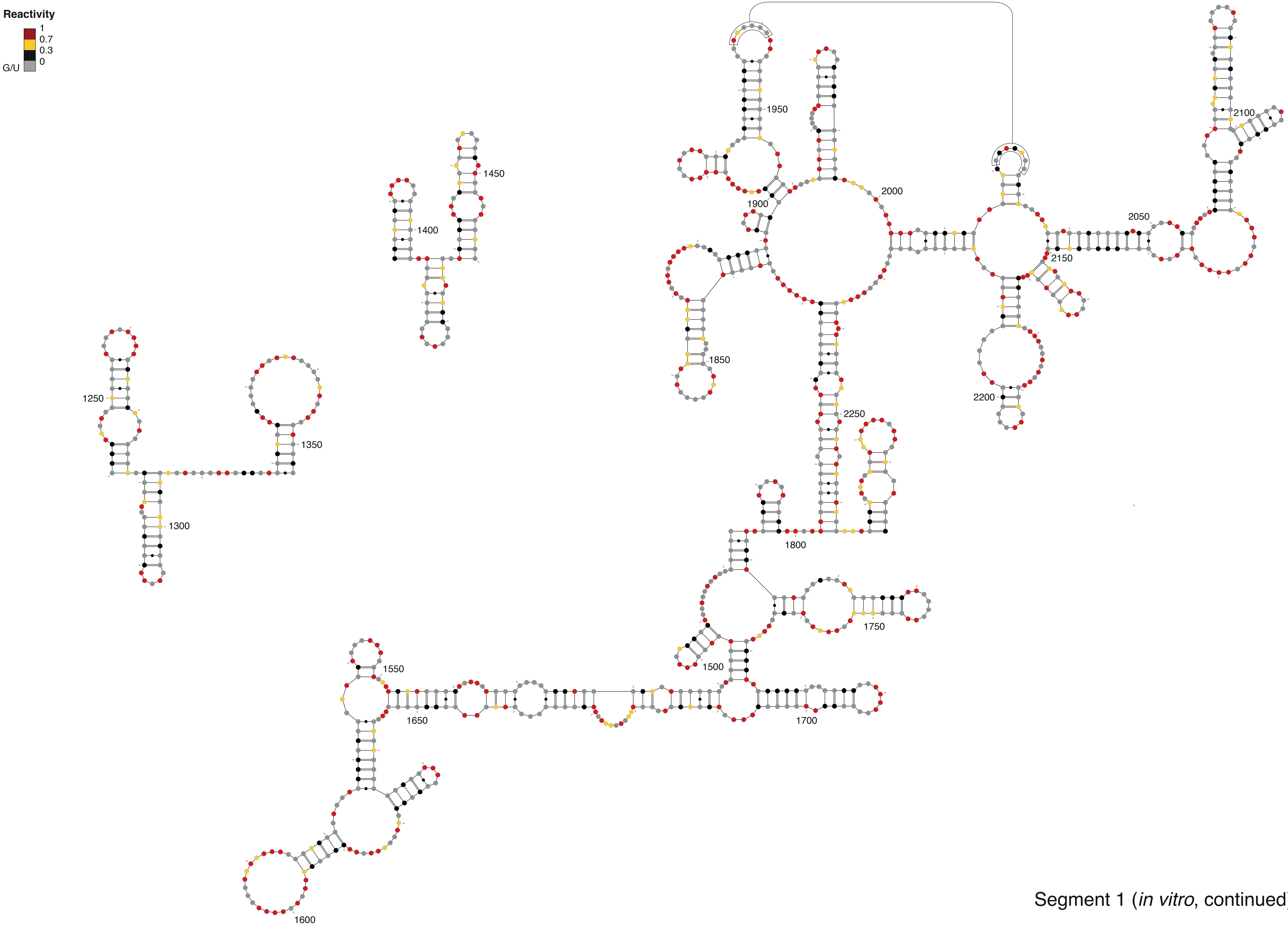

Segment 1 (*in vitro*, continued)

Figure S11. *In vitro* secondary structure model for IAV segment 1 (PB2) mRNA.

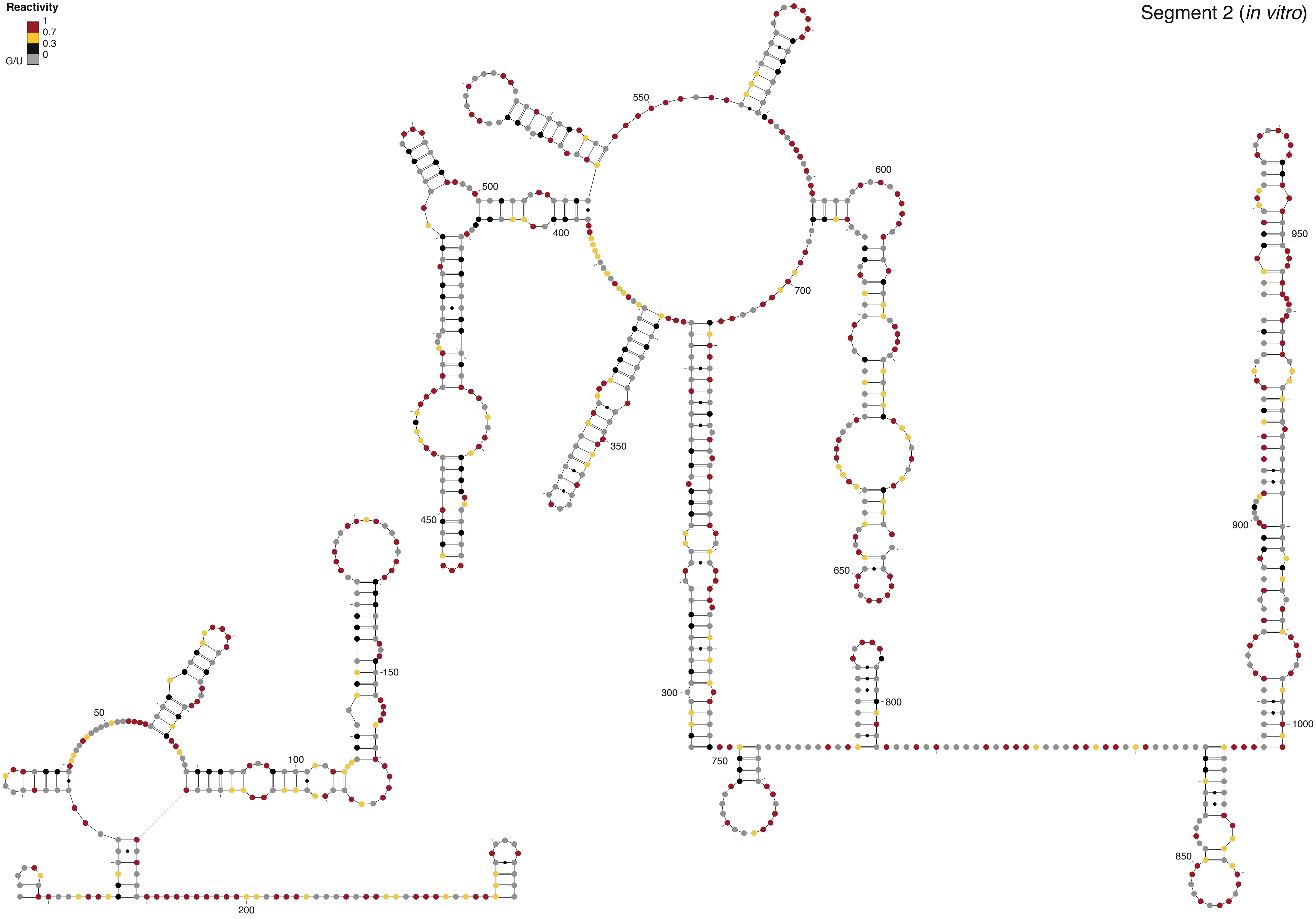
Reactivity
1
0.7
0.3
0
G/U
Segment 2 (*in vitro*)
50
100
150
200
300
350
400
450
500
550
600
650
700
750
800
850
900
950
1000

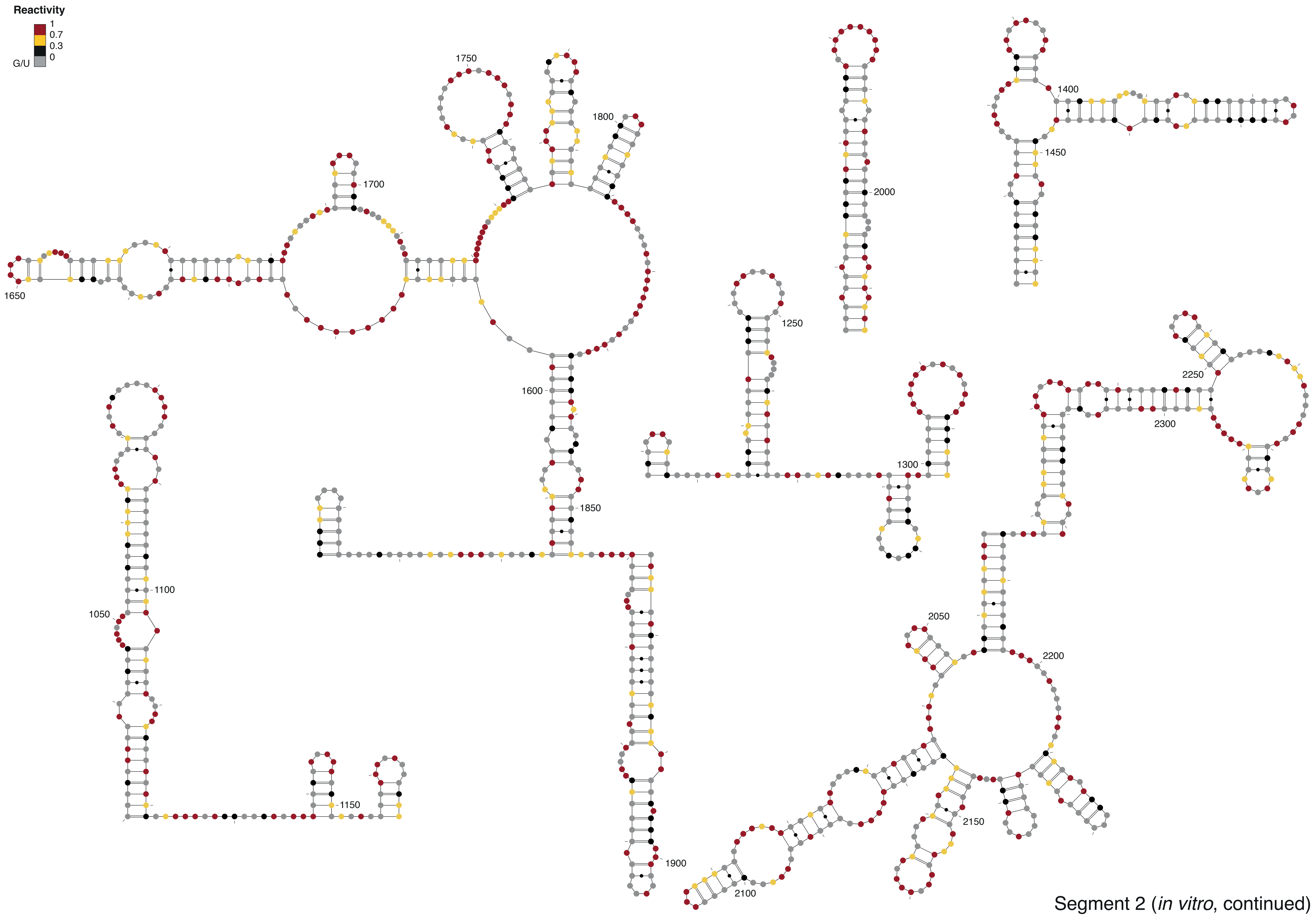

Segment 2 (*in vitro*, continued)

Figure S12. *In vitro* secondary structure model for IAV segment 2 (PB1) mRNA.

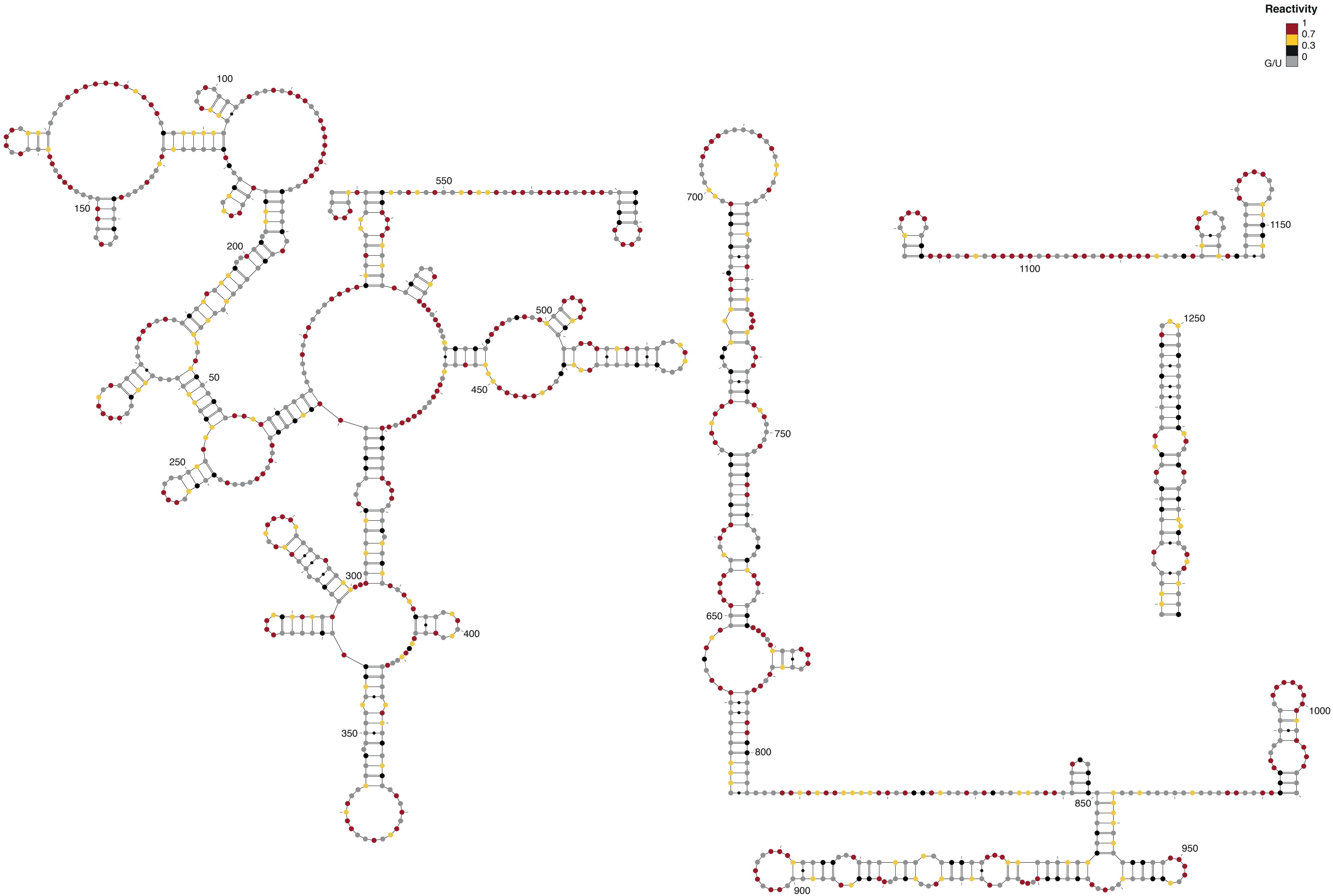

Segment 3 (*in vitro*)

Segment 3 (*in vitro*, continued)

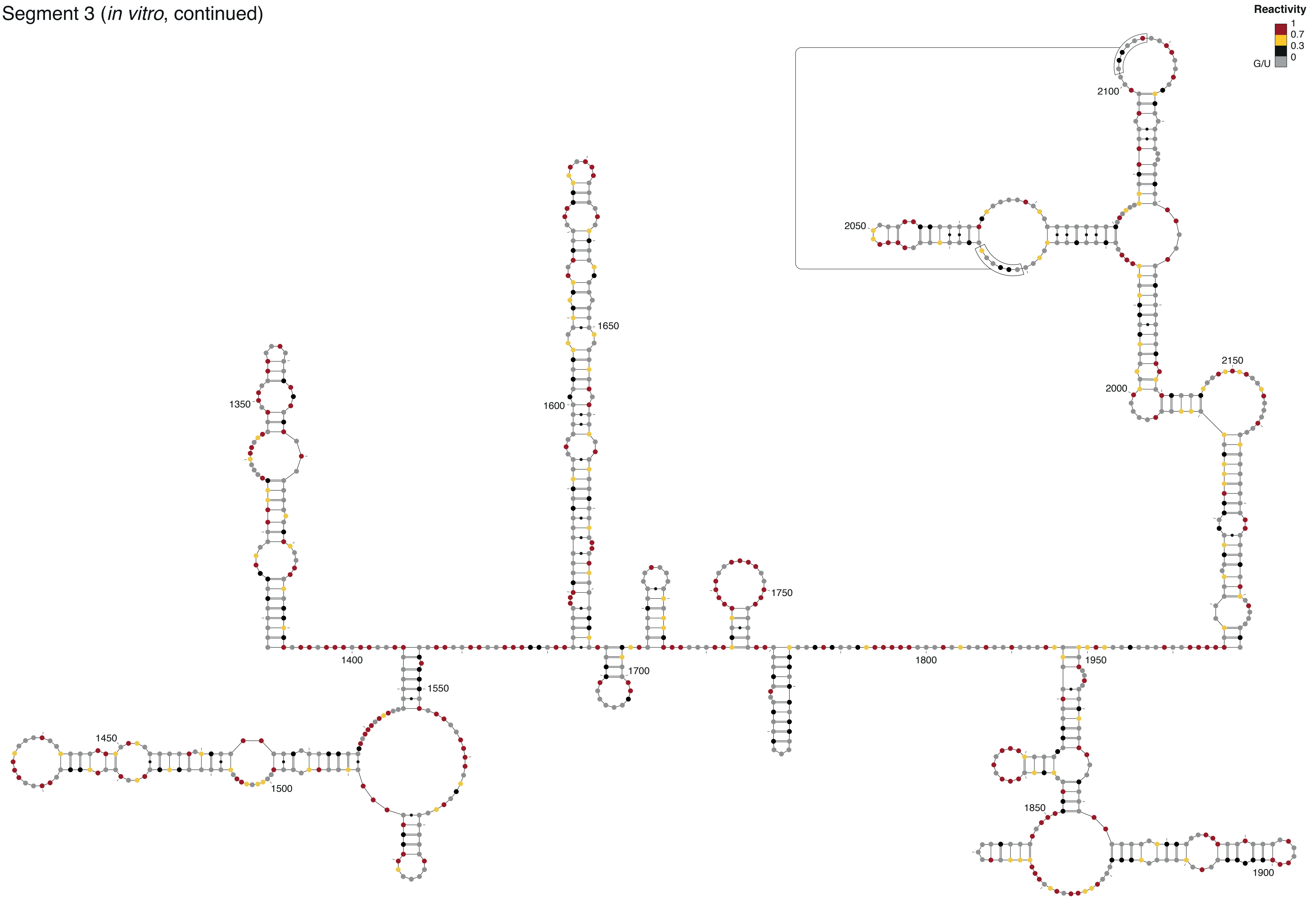

Figure S13. *In vitro* secondary structure model for IAV segment 3 (PA) mRNA.

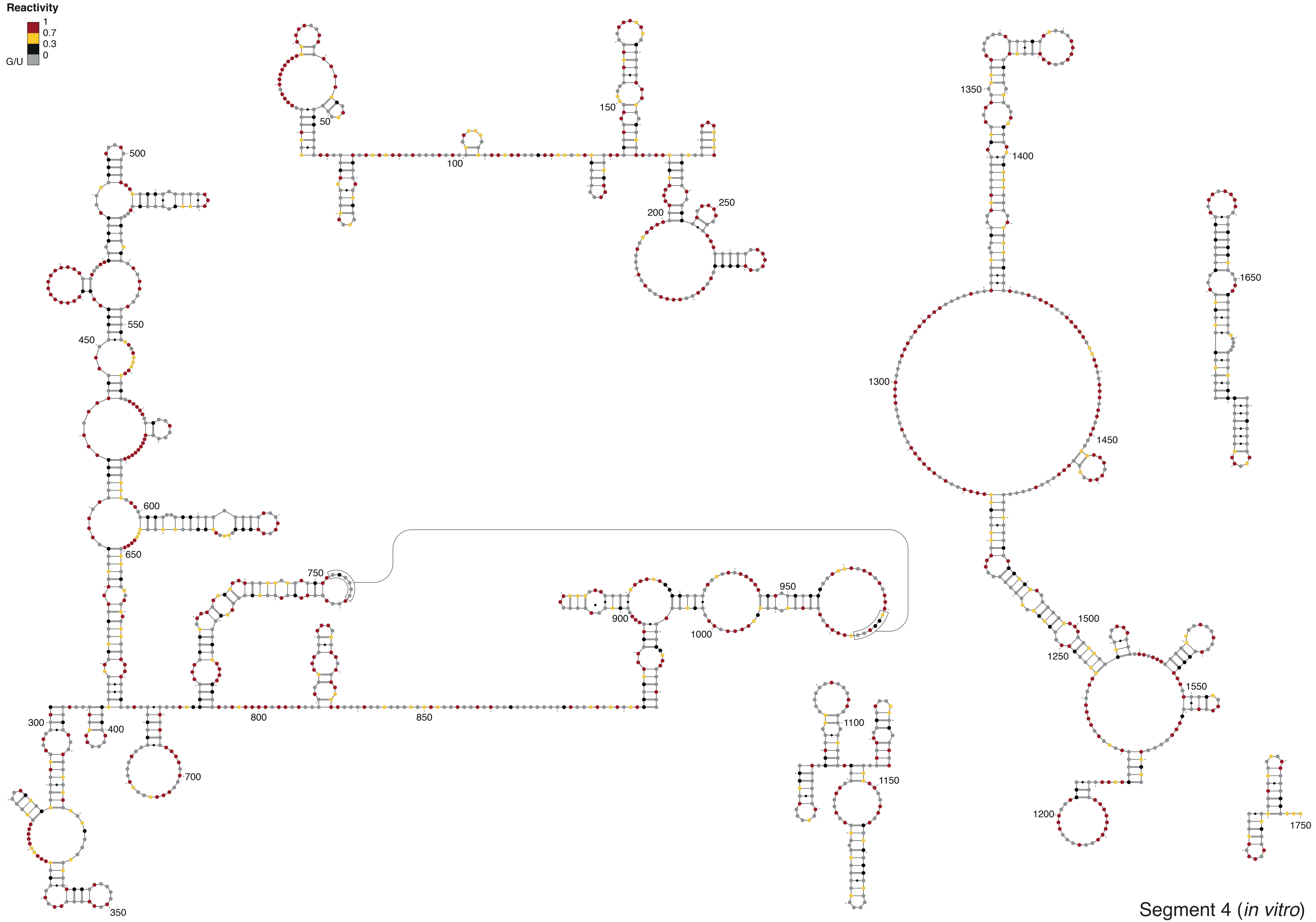

Segment 4 (*in vitro*)

Figure S14. *In vitro* secondary structure model for IAV segment 4 (HA) mRNA.

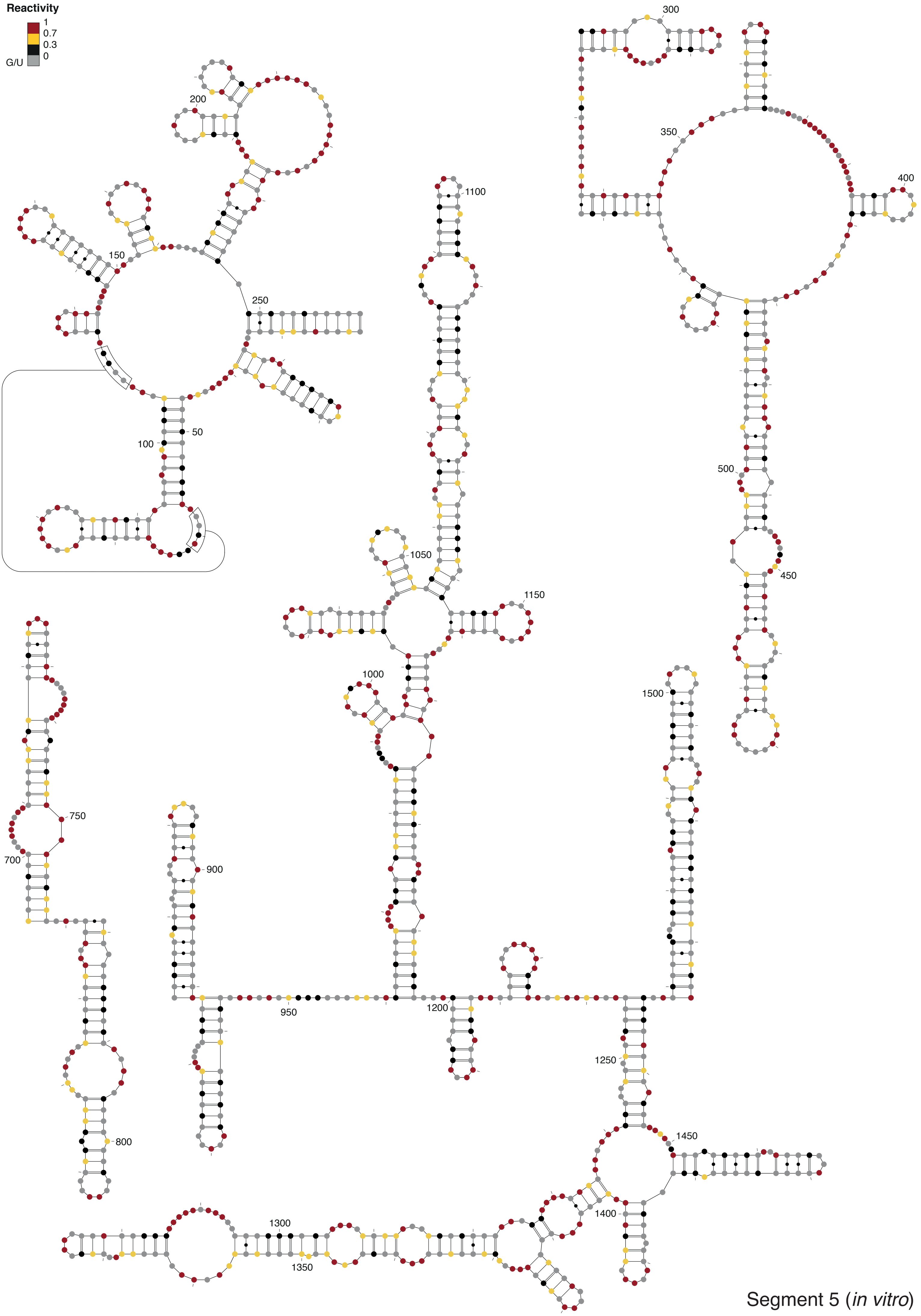

Segment 5 (*in vitro*)

Figure S15. *In vitro* secondary structure model for IAV segment 5 (NP) mRNA.

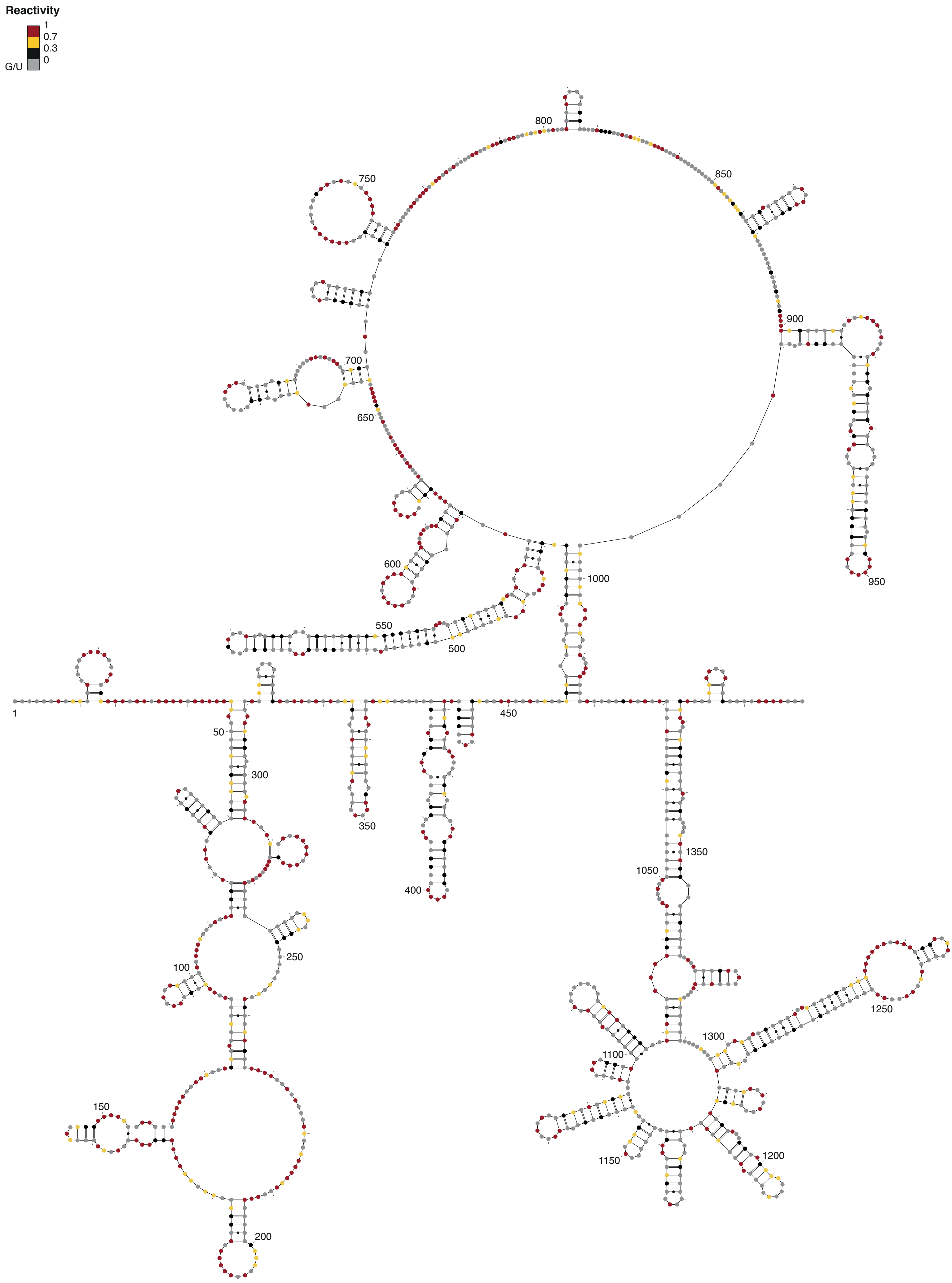

Segment 6 (*in vitro*)

Figure S16. *In vitro* secondary structure model for IAV segment 6 (NA) mRNA.

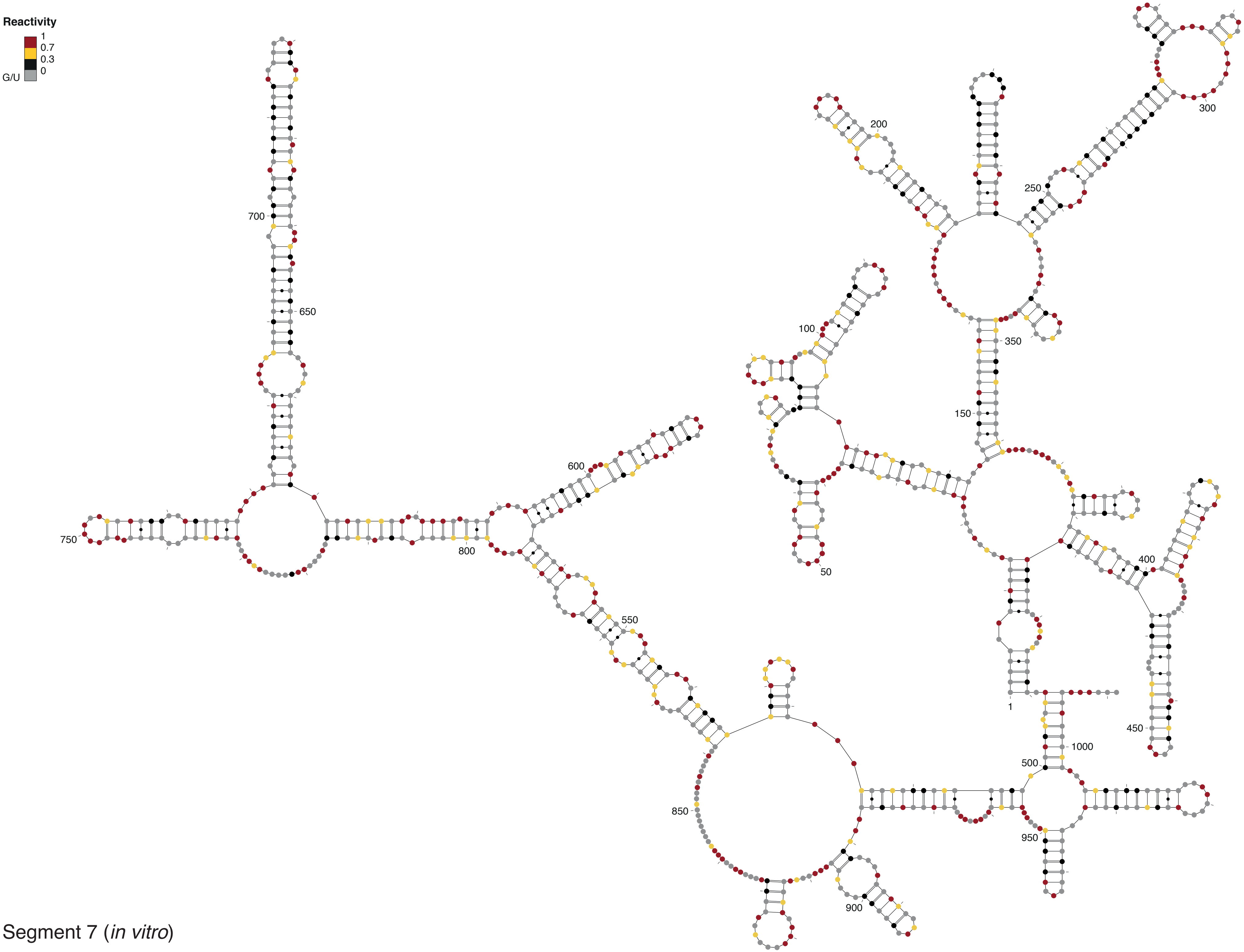

Segment 7 (*in vitro*)

Figure S17. *In vitro* secondary structure model for IAV segment 7, M (M1/M2) mRNA.

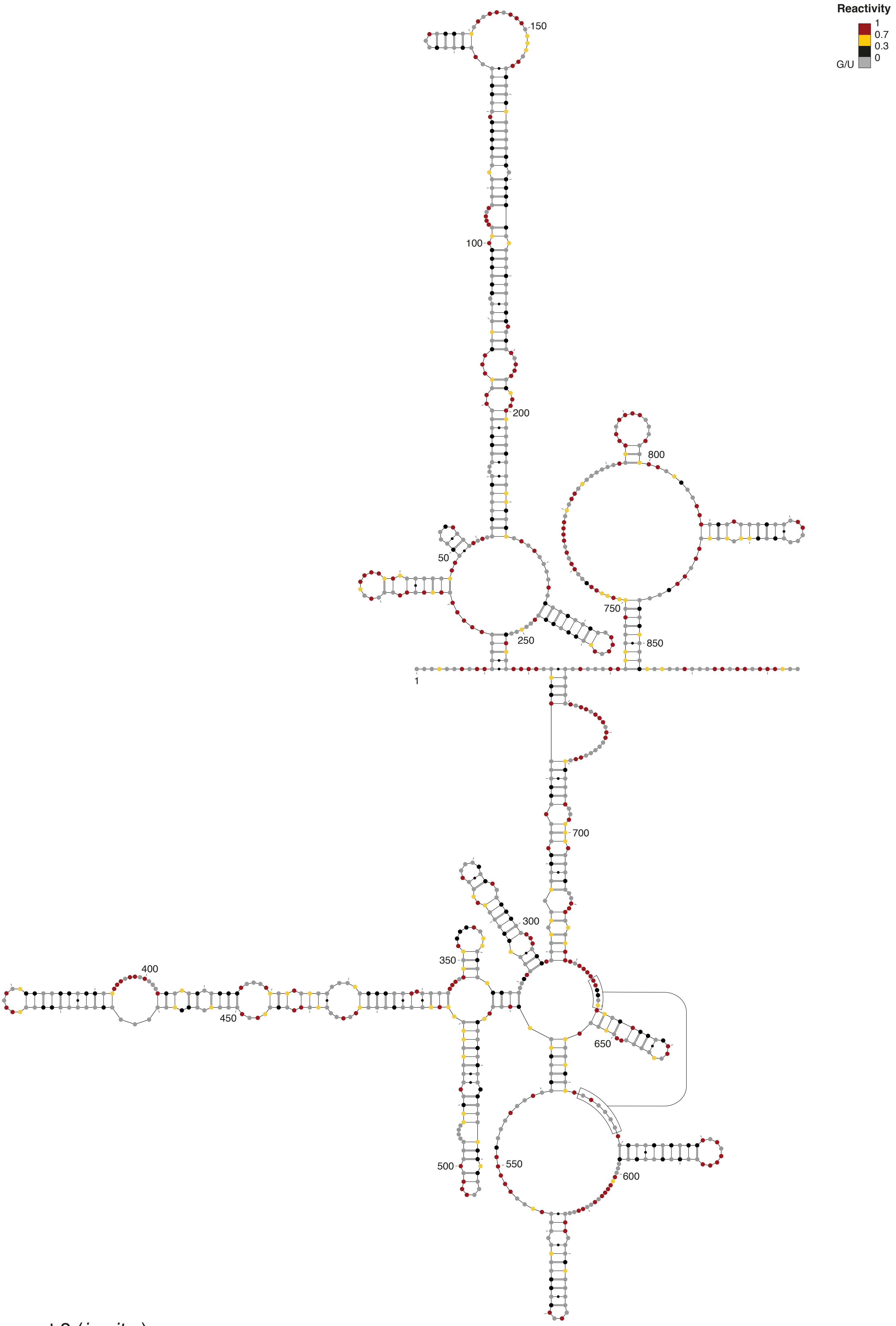

Segment 8 (*in vitro*)

Figure S18. *In vitro* secondary structure model for IAV segment 8, NS (NS1/NEP) mRNA.

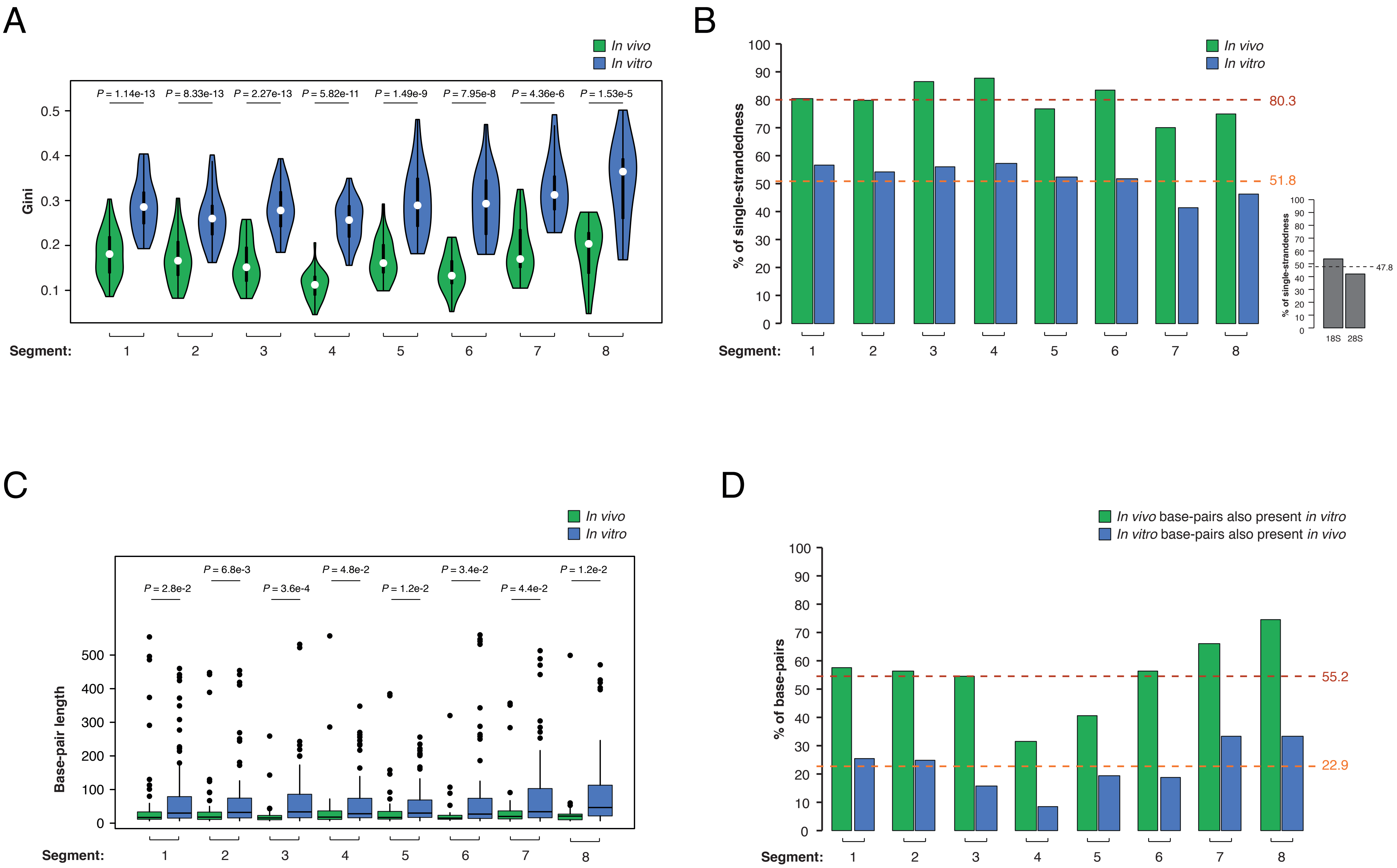

A
In vivo
In vitro
Gini
P = 1.14e-13
P = 8.33e-13
P = 2.27e-13
P = 5.82e-11
P = 1.49e-9
P = 7.95e-8
P = 4.36e-6
P = 1.53e-5
Segment:
B
% of single-strandedness
80.3
51.8
47.8
18S 28S
C
Base-pair length
P = 2.8e-2
P = 6.8e-3
P = 3.6e-4
P = 4.8e-2
P = 1.2e-2
P = 3.4e-2
P = 4.4e-2
P = 1.2e-2
D
In vivo base-pairs also present in vitro
In vitro base-pairs also present in vivo
% of base-pairs
55.2
22.9

Figure S19. (**A**) Violin plot of Gini indexes calculated on *in vivo* and *in vitro* 2-step normalized DMS reactivities, in non-overlapping windows containing 50 A/C residues. P-values are given by paired Wilcoxon test statistics, and adjusted by FDR. (**B**) Bar plot indicating the percentage of single-stranded residues in either the *in vivo* (green bars) or *in vitro* (blue bars) secondary structure models of IAV mRNAs. The red and orange dashed lines indicate respectively the median percentage of single-stranded residues in the *in vivo* and in the *in vitro* models. The percentage of single-stranded residues in mouse rRNAs is also indicated in the bottom-right corner for comparison. (**C**) Box plot of base-pair length distributions in *in vivo* (green) versus *in vitro* (blue) secondary structure models of IAV mRNAs. P-values are given by Wilcoxon test statistics, and adjusted by FDR. (**D**) Bar plot indicating the percentage of predicted base-pairs in the *in vivo* model that are also present in the *in vitro* model (green bars) or vice versa (blue bars). The red and orange dashed lines indicate respectively the median percentage of predicted base-pairs in the *in vivo* model that are also present in the *in vitro* model and vice versa.

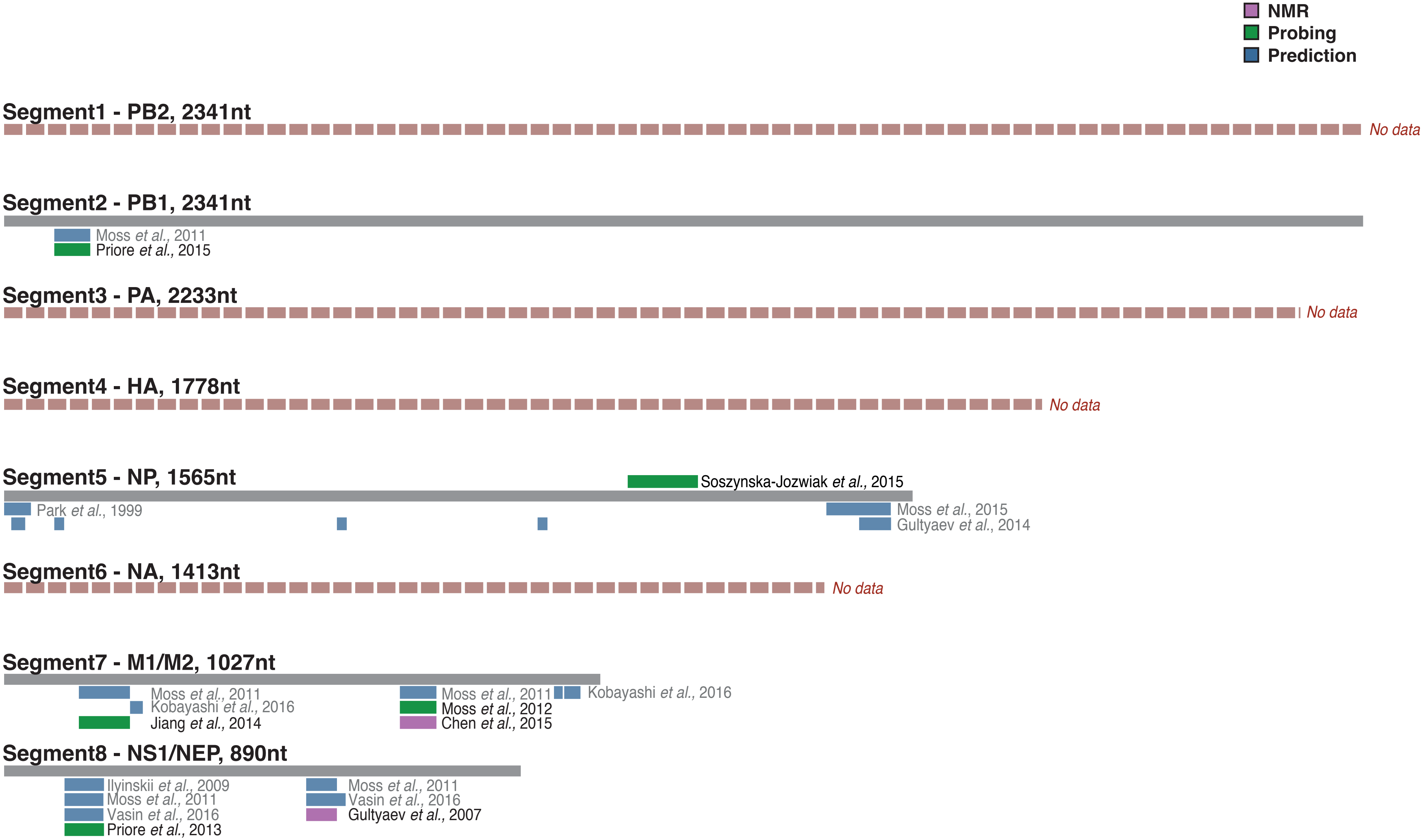

NMR
Probing
Prediction
Segment1 - PB2, 2341nt
No data
Segment2 - PB1, 2341nt
Moss et al., 2011
Priore et al., 2015
Segment3 - PA, 2233nt
No data
Segment4 - HA, 1778nt
No data
Segment5 - NP, 1565nt
Soszynska-Jozwiak et al., 2015
Park et al., 1999
Moss et al., 2015
Gultyaev et al., 2014
Segment6 - NA, 1413nt
No data
Segment7 - M1/M2, 1027nt
Moss et al., 2011
Kobayashi et al., 2016
Jiang et al., 2014
Moss et al., 2011
Moss et al., 2012
Chen et al., 2015
Kobayashi et al., 2016
Segment8 - NS1/NEP, 890nt
Ilyinskii et al., 2009
Moss et al., 2011
Vasin et al., 2016
Priore et al., 2013
Moss et al., 2011
Vasin et al., 2016
Gultyaev et al., 2007

Figure S20. Schematic map of literature-available IAV mRNA secondary structure data. Dashed lines indicate the lack of any structural information for the related segment.

In vitro	In vivo	Supported by DMS-MaPseq: In vitro	Supported by DMS-MaPseq: In vivo	Segment	Position	Reference
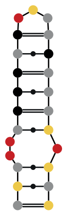	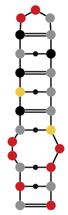	Yes	Partially	5	16	Park *et al.*, 1999<br>Gultyaev *et al.*, 2014
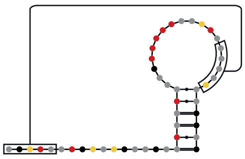	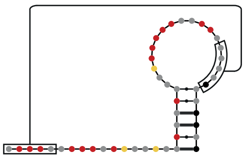	No	No	8	524	Gultyaev *et al.*, 2007<br>Moss *et al.*, 2011<br>Vasin *et al.*, 2016
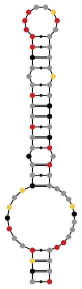	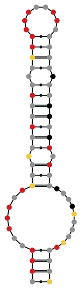	No	No			
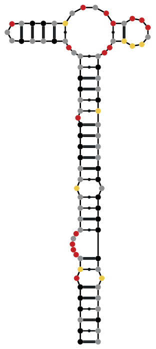	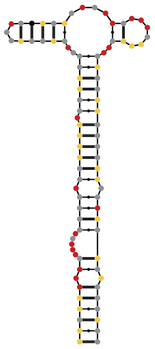	Yes	Partially	8	94	Ilyinskii *et al.*, 2009<br>Priore *et al.*, 2013
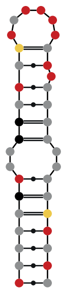	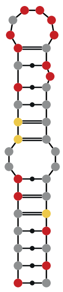	No	No	7	732	Moss *et al.*, 2011<br>Moss *et al.*, 2012<br>Chen *et al.*, 2015
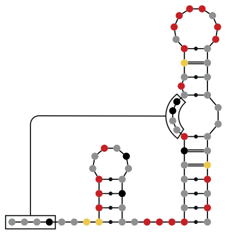	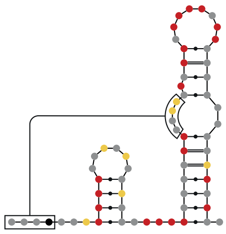	No	No		707	

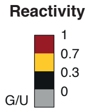

see next page

In vitro	In vivo	Supported by DMS-MaPseq: In vitro	Supported by DMS-MaPseq: In vivo	Segment	Position	Reference
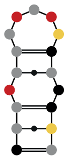	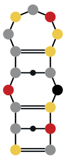	Yes	No	5	89	Gultyaev *et al.*, 2014
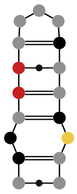	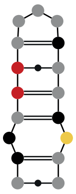	No	No	5	577	Gultyaev *et al.*, 2014
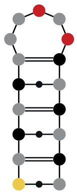	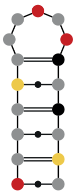	Yes	Yes	5	922	Gultyaev *et al.*, 2014
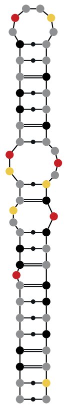	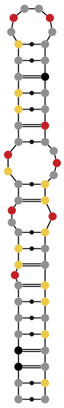	Yes	Partially	5	1476	Gultyaev *et al.*, 2014<br>Moss *et al.*, 2015
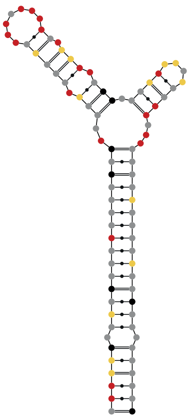	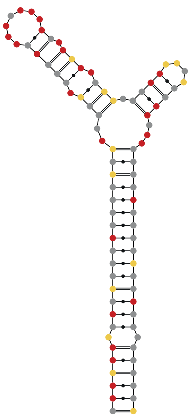	Partially	No	7	130	Moss *et al.*, 2011<br>Jiang *et al.*, 2014
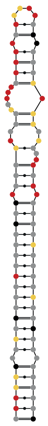	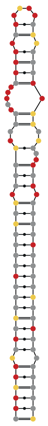	No	No			

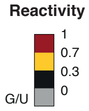

see next page

In vitro	In vivo	Supported by DMS-MaPseq: *In vitro*	Supported by DMS-MaPseq: *In vivo*	Segment	Position	Reference
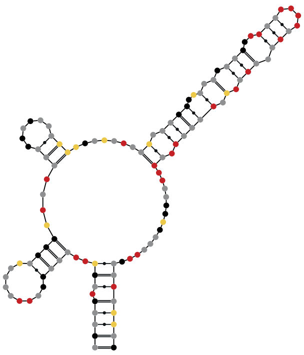	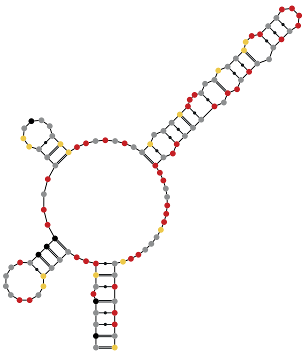	Partially	No	5	1079	Soszynska-Jozwiak *et al.*, 2015
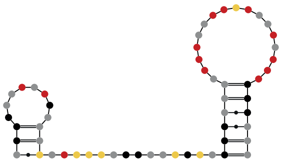	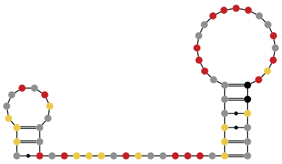	Yes	Yes	2	89	Moss *et al.*, 2011<br>Priore *et al.*, 2015
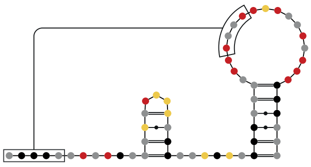	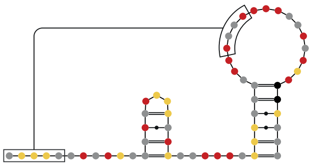	Yes	Partially			
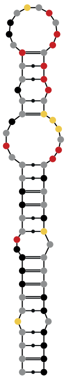	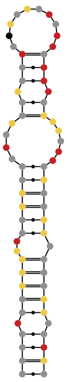	Partially	Partially	8	108	Moss *et al.*, 2011<br>Vasin *et al.*, 2016
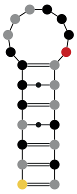	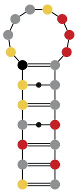	Partially	No	7	219	Kobayashi *et al.*, 2016
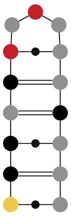	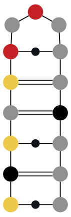	Yes	Yes	7	950	Kobayashi *et al.*, 2016
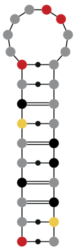	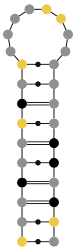	Yes	Yes	7	967	Kobayashi *et al.*, 2016

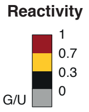

Figure S21. Summary table of literature-available IAV mRNA secondary structure models, with superimposed reactivities from this study. The compatibility of our DMS reactivity data with the respective proposed secondary structure models is also indicated. Compatibility was defined as follows: 1) structures having no highly reactive residues falling within double-stranded regions (excluding terminal pairs or pairs adjacent to loops/bulges) were marked as "Yes"; 2) structures having >0 and < 30% of highly reactive residues falling within double-stranded regions (excluding terminal pairs) or >50% of single-stranded low reactive residues, were marked as "Partially"; 3) Structures failing to match previous criteria were marked as "No".

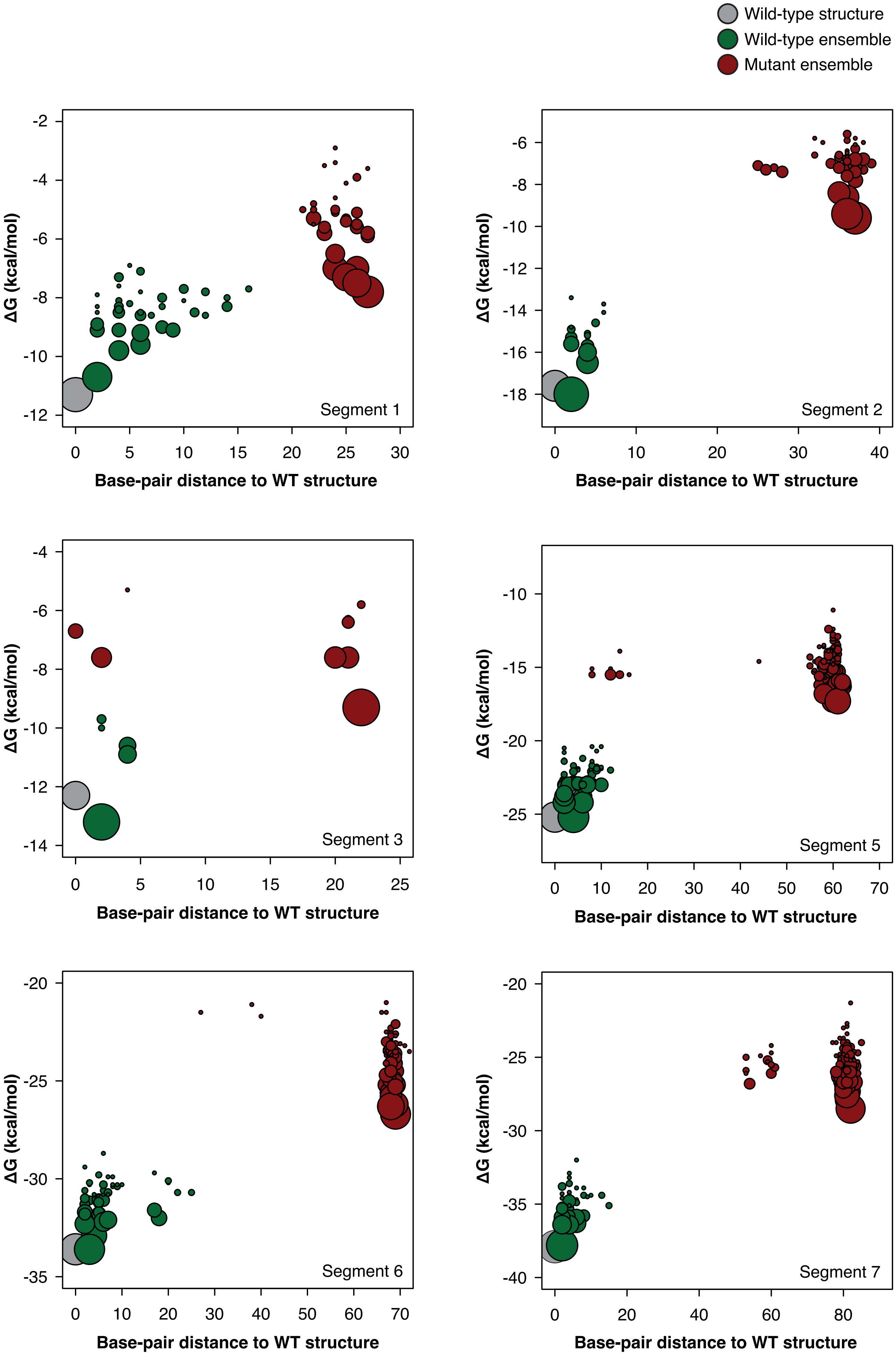
Wild-type structure
Wild-type ensemble
Mutant ensemble
ΔG (kcal/mol)
Base-pair distance to WT structure
Segment 1
Segment 2
Segment 3
Segment 5
Segment 6
Segment 7

Figure S22. *In silico* design of structure-disrupting mutants for the top-ranking RNA structural motifs of each IAV segment. Each circle corresponds to a different structure within the Boltzmann ensemble. The diameter of each circle corresponds to the $\log_2$ of the relative abundance of the respective structure within the ensemble. Free energies at 37 °C and base-pair distances were computed in the absence of any experimental constraint; thus, the predicted structure might slightly differ from the experimentally-determined structure.

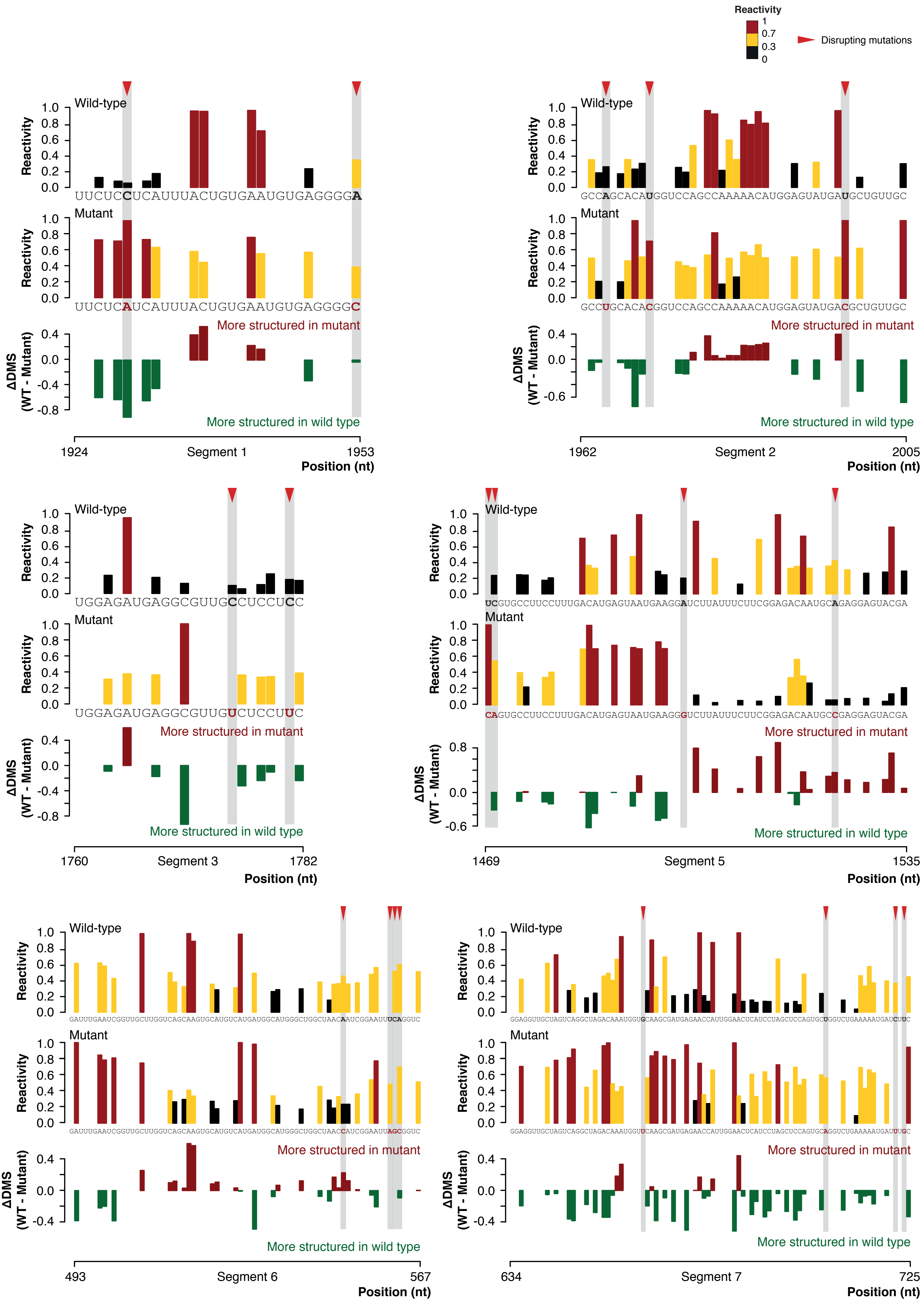

Reactivity
1
0.7
0.3
0
Disrupting mutations
Wild-type
Mutant
Reactivity
ΔDMS (WT - Mutant)
More structured in mutant
More structured in wild type
UUCUCCUCAUUUACUGUGAAUGUGAGGGGA
UUCUCAUCAUUUACUGUGAAUGUGAGGGGC
1924
Segment 1
1953
Position (nt)
GCCAGCACAUGGUCCAGCCAAAAACAUGGAGUAUGAUGCUGUUGC
GCCUGCACACGGUCCAGCCAAAAACAUGGAGUAUGACGCUGUUGC
1962
Segment 2
2005
UGGAGAUGAGGCGUUGCCUCCUCC
UGGAGAUGAGGCGUUGUCUCCUUC
1760
Segment 3
1782
UCGUGCCUUCCUUUGACAUGAGUAAUGAAGGAUCUUAUUUCUUCGGAGACAAUGCAGAGGAGUACGA
CAGUGCCUUCCUUUGACAUGAGUAAUGAAGGGUCUUAUUUCUUCGGAGACAAUGCCGAGGAGUACGA
1469
Segment 5
1535
GAUUUGAAUCGGUUGCUUGGUCAGCAAGUGCAUGUCAUGAUGGCAUGGGCUGGCUAACAAUCGGAAUUUCAGGUC
GAUUUGAAUCGGUUGCUUGGUCAGCAAGUGCAUGUCAUGAUGGCAUGGGCUGGCUAACCAUCGGAAUUAGCGGUC
493
Segment 6
567
GGAGGUUGCUAGUCAGGCUAGACAAAUGGUGCAAGCGAUGAGAACCAUUGGAACUCAUCCUAGCUCCAGUGCUGGUCUGAAAAAUGAUCUUC
GGAGGUUGCUAGUCAGGCUAGACAAAUGGUUCAAGCGAUGAGAACCAUUGGAACUCAUCCUAGCUCCAGUGCAGGUCUGAAAAAUGAUUUGC
634
Segment 7
725

Figure S23. Targeted DMS-MaPseq analysis of the mutated top-ranking RNA structural motif of each IAV segment. 90% Winsorizing-normalized reactivities are reported.
